# Supplementary material for: Cross-sectional study of COVID-19 knowledge, beliefs and prevention behaviours among adults in Senegal
Source: BMJ Open. 2022 May 26;12(5):e057914. doi: 10.1136/bmjopen-2021-057914 (PMC9136694; doi:10.1136/bmjopen-2021-057914)
Supplement: Supplementary data [file bmjopen-2021-057914supp001.pdf]

Supplemental Table 1. Sample characteristics stratified by recruitment method. N=1,452. Chi-squared and paired t-tests assessed differences between methods. Alpha=0.05. Column percentages presented. Data collected via phone and online surveys from Senegalese adults between June-August 2020.

**Characteristic**	**Category**	**Phone**	**Online**	**Total**	***p***
Age (Mean/Median)		28.5/27.0	25.0/23.0	28.0/26.0	<.001
Age Quartile	<21	23.1	30.7	24.3	<.001
	22-25	22.3	34.6	24.2	
	26-34	23.9	26.8	24.4	
	35+	30.7	7.8	27.1	
Sex	Female	50.1	60.6	51.8	0.003
	Male	49.9	39.4	48.2	
Location	Urban	74.7	89.6	77.1	<.001
	Rural	23.0	4.4	20.1	
	Urban + Rural	2.3	6.1	2.9	
Education	Superior	16.1	79.7	26.2	<.001
	Secondary	25.1	16.0	23.7	
	Primary	23.3	0.0	19.6	
	Middle	18.6	1.7	15.9	
	No	11.8	0.4	10.0	
	Other	5.1	2.2	4.6	
Marital status	Single	50.9	78.8	55.4	<.001
	Married - monogamous	34.9	14.7	31.7	
	Married - polygamist	12.5	3.9	11.1	
	Divorced	1.2	2.2	1.4	
	Widower	0.5	0.4	0.5	
Occupation	Informal sector	45.6	5.2	39.2	<.001
	Student	20.0	67.5	27.6	
	Formal sector	14.6	20.4	15.5	
	Other	8.4	3.9	7.7	
	Housewife	7.5	2.2	6.6	
	Retired, out of work	3.9	0.9	3.4	
	Religious leader	0.1	0.0	0.1	
Religion	Muslim	95.9	87.0	94.5	<.001
	Catholic	3.9	7.8	4.5	
	Protestant / Evangelical	0.1	3.0	0.6	
	No	0.0	1.3	0.2	
	Other	0.1	0.9	0.2	
	Traditionalist	0.1	0.0	0.1	

Supplemental Table 2. Descriptive statistics for novel COVID-19 subscales on knowledge & awareness (n=1,447), perceived threat (n=1,445), prevention behaviors (n=1,447), and all subscales combined (n=1,441). Subscales ranged from 0 [min] to 15 [max]; total scale ranged from 0 to 45. Data collected via phone and online surveys from Senegalese adults between June-August 2020.

	Mean	SD	Median
Knowledge & awareness	11.8	2.6	12.5
Perceived threat	10.9	3.5	11.7
Prevention behaviors	10.5	2.3	11.0
Total	33.1	6.0	34.0
