# Supplementary material for: Cross-sectional study of COVID-19 knowledge, beliefs and prevention behaviours among adults in Senegal
Source: BMJ Open. 2022 May 26;12(5):e057914. doi: 10.1136/bmjopen-2021-057914 (PMC9136694; doi:10.1136/bmjopen-2021-057914)
Supplement: Author's manuscript [file bmjopen-2021-057914.draft_revisions.pdf]

BMJ Open

BMJ Open is committed to open peer review. As part of this commitment we make the peer review history of every article we publish publicly available.

When an article is published we post the peer reviewers' comments and the authors' responses online. We also post the versions of the paper that were used during peer review. These are the versions that the peer review comments apply to.

The versions of the paper that follow are the versions that were submitted during the peer review process. They are not the versions of record or the final published versions. They should not be cited or distributed as the published version of this manuscript.

BMJ Open is an open access journal and the full, final, typeset and author-corrected version of record of the manuscript is available on our site with no access controls, subscription charges or pay-per-view fees (http://bmjopen.bmj.com).

If you have any questions on BMJ Open's open peer review process please email info.bmjopen@bmj.com

BMJ Open

A cross-sectional study of COVID-19 knowledge, beliefs and prevention behaviors among adults in Senegal

Journal:	*BMJ Open*
Manuscript ID	bmjopen-2021-057914
Article Type:	Original research
Date Submitted by the Author:	11-Oct-2021

Complete List of Authors:	Kearney, Matthew; University of Pennsylvania Perelman School of Medicine, Family Medicine and Community Health<br>Bornstein, Marta; The Ohio State University<br>Fall, Marieme; The African Health and Education Network (NGO RAES)<br>Nianogo, Roch; UCLA School of Public Health, Epidemiology<br>Glik, D; UCLA, Community Health Sciences<br>Massey, Philip; University of Arkansas Fayetteville, Health, Human Performance, and Recreation

Keywords:	COVID-19, Public health < INFECTIOUS DISEASES, PUBLIC HEALTH

SCHOLARONE™
Manuscripts

I, the Submitting Author has the right to grant and does grant on behalf of all authors of the Work (as defined in the below author licence), an exclusive licence and/or a non-exclusive licence for contributions from authors who are: i) UK Crown employees; ii) where BMJ has agreed a CC-BY licence shall apply, and/or iii) in accordance with the terms applicable for US Federal Government officers or employees acting as part of their official duties; on a worldwide, perpetual, irrevocable, royalty-free basis to BMJ Publishing Group Ltd ("BMJ") its licensees and where the relevant Journal is co-owned by BMJ to the co-owners of the Journal, to publish the Work in this journal and any other BMJ products and to exploit all rights, as set out in our licence.

The Submitting Author accepts and understands that any supply made under these terms is made by BMJ to the Submitting Author unless you are acting as an employee on behalf of your employer or a postgraduate student of an affiliated institution which is paying any applicable article publishing charge ("APC") for Open Access articles. Where the Submitting Author wishes to make the Work available on an Open Access basis (and intends to pay the relevant APC), the terms of reuse of such Open Access shall be governed by a Creative Commons licence – details of these licences and which Creative Commons licence will apply to this Work are set out in our licence referred to above.

Other than as permitted in any relevant BMJ Author's Self Archiving Policies, I confirm this Work has not been accepted for publication elsewhere, is not being considered for publication elsewhere and does not duplicate material already published. I confirm all authors consent to publication of this Work and authorise the granting of this licence.

For peer review only

Title

A cross-sectional study of COVID-19 knowledge, beliefs and prevention behaviors among adults in Senegal

Authors

Matthew D. Kearney PhD MPH – Department of Family Medicine and Community Health, Perelman School of Medicine, University of Pennsylvania, Philadelphia, Pennsylvania, USA, ORCID ID: 0000-0003-2898-573X

Marta Bornstein PhD MPH – College of Public Health, The Ohio State University, Columbus, OH, USA, ORCID ID: 0000-0002-5200-8181

Marieme Fall MPH – African Health and Education Network (RAES), Dakar, Senegal

Roch Nianogo PhD – Department of Epidemiology, UCLA Fielding School of Public Health, Los Angeles, CA, USA, ORCID ID: 0000-0001-5932-6169

Deborah Glik PhD – Department of Community Health Sciences, UCLA Fielding School of Public Health, Los Angeles, CA, USA, ORCID ID: 0000-0002-2532-3661

Philip M. Massey PhD MPH – Department of Health, Human Performance, and Recreation, College of Education and Health Professions, University of Arkansas, Fayetteville, AR, USA, ORCID: 0000-0002-0577-8618

Corresponding Author

Name: Dr. Matthew D. Kearney

Address: Penn Presbyterian Medical Center, Andrew Mutch Building, 51 N. 39th Street, Philadelphia, PA 19104

Email: kearnm@pennmedicine.upenn.edu

Word Count: 4,034

For peer review only

Abstract

Objectives: The aim of the study was to explore COVID-19 beliefs and prevention behaviors in francophone West Africa.

Design: This was a secondary analysis of survey data collected via a multi-modal cross-sectional observational study.

Participants: Senegalese adults aged 18 years or older.

Primary and secondary outcome measures: Primary outcome measures were COVID-19 prevention behaviors. Secondary outcome measures included COVID-19 knowledge and beliefs. Univariate, bivariate, and multivariate statistics were generated to describe the sample and explore potential correlations.

Setting: Participants from Senegal were recruited online and telephonically between June-August 2020.

Results: Mask wearing, hand washing, and use of hand sanitizer were most frequently reported. Social distancing and staying at home were also reported albeit to a lower degree. We also identified a range of psychosocial and demographic predictors for COVID-19 prevention behaviors. Men, compared to women, had lower odds (AOR=0.59, 95% CI: 0.46-0.75, p<.001) of reporting prevention behaviors. Rural residents (vs. urban; AOR=1.49, 95% CI: 1.12-1.98, p=.001) and participants with at least a high school education (vs. less than high school education; AOR=1.33, 95% CI: 1.01-1.76, p=.006) were more likely to report COVID-19 prevention behaviors.

Conclusions: Stakeholders and decision makers in Senegal and across Africa can use place-based evidence like ours to address COVID-19 risk factors and intervene

For peer review only - http://bmjopen.bmj.com/site/about/guidelines.xhtml

effectively with policies and programming. Use of both phone and online surveys enhances representation and study generalizability and should be considered in future research with hard-to-reach populations.

For peer review only

Article Summary

Strengths and limitations of this study

- The main strength of our study is the use of a multi-modal data collection strategy, online and via telephone. Had we relied on a single method, our sample's demographic characteristics would likely have differed while also introducing selection bias.
- Our recruitment strategy may have also increased the potential for selection bias because participants were recruited online and on-the-ground in Senegal; thus, all participants, by the nature of the recruitment methods, had access to the internet and/or a cell phone. To address potential confounding between recruitment methods, we controlled for recruitment method in our multivariate regression modelling.
- Prior to our study, little evidence was available about how populations in francophone West Africa have responded to COVID-19 in their daily lives. We adapted pre-existing research panels and developed novel data collection instruments to capture information about COVID-19, and this may serve as a model for the responsiveness of ongoing scholarship to future global events.
- A study with a larger sample may have been able to identify relationships between knowledge and behaviors, and our study was not design with enough statistical power to detect significant differences.
- The findings of our study may not be generalizable beyond Senegal, or more specifically Senegalese adults.

For peer review only

Introduction

COVID-19 is an ongoing threat to global public health since its emergence at the end of 2019, yet little is known about how populations in low-resource settings have responded to COVID-19 in their daily lives. Although research is emerging about COVID-19 infection, strategies for prevention, and risk factors for severe disease and mortality, there is a dearth of literature about COVID-19 beliefs, specifically in the West Africa region. For example, between January and September of 2020, just 4.3% of research articles about COVID-19 were focused on Africa or a specific African nation.[1] Given that ongoing prevention strategies implemented at the country, community, and individual level will be needed to curb the COVID-19 pandemic, it is critical to study health beliefs and adherence to prevention behaviors within specific social and political contexts.[2]

Providing relevant and timely research is essential for guiding evidence-based outreach and interventions, especially during public health emergencies. It is particularly important to advance equity within public health, and the health of populations, by understanding the social contexts and health beliefs among indigenous, marginalized, and vulnerable populations around the globe.[3,4] The current study provides timely, critical, and actionable information about COVID-19 beliefs and prevention behaviors for stakeholders, policy makers, and public health programs to reduce the burden of COVID-19 in West Africa by reporting on findings among a sample of adults from the West African nation of Senegal.

For peer review only

West African Context

West Africa is a distinct language and cultural region made up of 16 sovereign nations, many of which were formerly French and British colonies. Increasing internet infrastructure and use of mobile technologies has bridged historical digital disparities and inequities by allowing unprecedented access to health information for many peoples living in West Africa and other developing regions.[5–7] Francophone West Africa, in particular, is underrepresented in global health research because of language barriers to publication and collaboration, leading to more research in English-speaking nations like Nigeria and Ghana.[8–10] Increasing global internet connectivity has also augmented representation of formerly hard-to-reach vulnerable and marginalized populations in the research process as both participants and collaborators.[8–10]

Senegal is the former capital of French West Africa and home to over 16 million people. Approximately 90% of the population is Muslim and 5% are Christian, most of whom are Catholic.[11] Senegalese culture and politics are infused with both French and Islamic influences, and Senegal is often considered a model democracy. Although Senegal has a thriving tourism industry and developed financial services and international trade sectors, the nation still ranks low in the United Nation's Human Development Index, 35th out of the 53 ranked African nations.[12]

COVID-19 in West Africa

Compared to the burden of COVID-19 in Europe, the Americas, the Middle East and South Asia, the burden of COVID-19 in Africa has been quite low. Relative to the rest of the world, Africa defied initial expectations by experiencing the lowest burden of reported COVID-19 cases and deaths.[13,14] Questions remain about why COVID-19

For peer review only

infections are reportedly low in Africa, in particular in West Africa where there have been 377 cases per 100,000 persons cumulatively. Although a recent analysis by Aduh and colleagues (2020) reported on perceptions and behaviors towards COVID-19 across Sub-Saharan Africa, we have much to learn from populations in densely populated West Africa, a comparatively more urban Sub-Saharan region home to more than 400 million people.[16]

Some researchers have questioned the accuracy of African COVID-19 case numbers,[13] hypothesizing that the burden of disease could be much higher than reported. Uncertainty about the true trajectory of COVID-19 in Africa may leave all continents vulnerable to future outbreaks, particularly regions with low herd immunity due to lack of exposure and vaccine resource constraints. It is likely that places that that have not yet needed to adopt strong prevention efforts because of reportedly low case numbers therefore may be especially vulnerable to future outbreaks. Thus, despite relatively low case numbers, it is still vital to investigate COVID-19 health beliefs and prevention behaviors in West Africa peoples to identify potential correlates and predictors of viral prevention behaviors.

Study Objectives

To explore COVID-19 beliefs and prevention behaviors in francophone West Africa, the current study analyzes survey data collected from a sample of adults in Senegal. The survey, which was administered both online and via telephone, included a module on COVID-19 behaviors and beliefs. Specifically, this study applies constructs from the Health Belief Model to answer the following questions:[17,18]

1) In Senegal, what is the level of knowledge and awareness about COVID-19?

For peer review only

2) To what extent is COVID-19 perceived as a health threat?

3) What is the relationship between COVID-19 beliefs and prevention behaviors?

Methods

Study Design, Participants and Setting

From June-August 2020, we conducted a multi-modal cross-sectional survey that included questions regarding COVID-19 beliefs and prevention behaviors among adults in Senegal. The survey was administered via the internet and telephone, which was appropriate given the constraints of research during COVID-19. Respondents were participating in a separate ongoing longitudinal cohort study.

Data Collection

This survey used two modes of data collection: online and telephone. The telephone survey used a random selection from a panel of phone numbers of individuals who lived in Senegal and agreed to participate in media-related research. A quota system was used to match the overall population of Senegal on gender and have an even distribution of age and level of education. A total of 1,221 participants were enrolled in the study via telephone, split approximately equally between participants living insider versus outside of Dakar, Senegal and by gender. Online participants from Senegal were recruited using social media advertisements on Facebook and YouTube. All online participants were part of a cohort study about entertainment education in West Africa that began in late summer 2019; of the 1,286 cohort participants recruited online in summer 2019, 231 both were eligible to participate (i.e., Senegalese adults) and

For peer review only - http://bmjopen.bmj.com/site/about/guidelines.xhtml

completed the COVID-19 questionnaire items. Through our multi-modal strategy, responses about COVID-19 were collected from 1,452 Senegalese adults.

For both online and telephone participants, all individuals were required to be 18 years or older. Potential participants were informed about the study and provided consent to participate in the survey (online) or interview (telephone). Consenting participants were compensated with 2000 Central African CFA franc (~4 USD) for their time. Incomplete responses with missing data were excluded. All study procedures and protocols were reviewed by and approved or determined to be exempt by institutional ethical review boards.

Measurement

Survey items included a series of self-reported demographic questions (e.g., age, education) as well as a 15-item section specifically about COVID-19 (see Appendix A). The COVID-19 section contained three measurement domains: 1) knowledge & awareness, 2) perceived threat to health, and 3) prevention behaviors. COVID-19 items were developed based on available guidance from the WHO and CDC and were reviewed by French, English, and Wolof speaking team members. Response options were binary (e.g., agree/disagree) or Likert-style (i.e., ordinal). Our data collection instrument drew upon the Health Belief Model (HBM),[18] a behavior change framework for public health practice positing that knowledge of a potential negative outcome and perceived threat of that outcome precede behavior change.[17] Other recent studies have similarly relied on HBM constructs to explore COVID-19.[19–21] COVID-19 response items were aggregated into distinct scales based on the three measurement domains. Each scale ranged from 0 to 15. As the COVID-19 items were novel, these

For peer review only - http://bmjopen.bmj.com/site/about/guidelines.xhtml

“scales” were used as a proxy measure for determining the degree of knowledge and awareness, perceived risk, and practicing of known prevention behaviors including 1) handwashing, 2) using hand sanitizer, 3) wearing a mask, 4) social distancing, and 5) staying at home.

Data Analysis

Univariate and bivariate statistics were generated to describe the sample and explore potential correlations. Chi-squared tests assessed differences in demographic attributes between the online and telephone collection methods. We used Pearson’s correlation coefficient to explore relationships between items in the COVID-19 scales. For multivariate analysis, we recoded level of education, marital status, location (e.g., urban/rural), religion, and occupation to reduce the number of categories, creating binary or ordinal variables, as shown in Table 1. Participant age was collected as a continuous measure and recoded as both quartile age groups and as binary (above versus below median age). Median values were calculated for each of the three COVID-19 scales and used as thresholds to create binary variables (e.g., 0=low knowledge;1=high knowledge).

We built five logistic regression models that predicted COVID-19 behaviors individually (i.e., hand washing, mask wearing, social distancing, staying home) and in aggregate, the results of which are presented as Forest plots (Figure 1). For individual behavior models, dependent variables were whether or not participants practiced prevention behaviors daily in the past week; for the aggregate model the dependent variable was the prevention behavior scale ranging from 0 (practice no behaviors in past week) to 15 (practice all behaviors daily). Independent variables in each model included

For peer review only

demographic items, perceived threat (high/low), and knowledge & awareness (high/low). All statistical analyses were conducted using Stata IC software (version 15). Forest plots were generated using the 'forestplot' package in RStudio software.[22]

Patient and Public Involvement

This study is a cross-sectional analysis of surveys conducted in 2020 using a multi-modal recruitment strategy. No patients were involved in our study. All analyses were conducted in collaboration with research partners in Dakar, Senegal so it was not appropriate to involve specific public communities or patients or in the design, conduct, reporting or dissemination plans.

Results

A total of 1,452 Senegalese adults participated in our cross-sectional survey about COVID-19 (telephone=1,221; online=231). Table 1 presents demographics characteristics for the study sample. Table 2 presents univariate descriptive statistics for the three COVID-19 scales. Figure 1 presents a Forest plot of our logistic regression model predicting prevention behaviors.

Sample Characteristics

Most participants identified as Muslim (94.5%) and primarily lived in in an urban area (77.1%). Nearly half completed secondary education (high school or more; 49.9%). Most participants were single (55.4%). Participants' ages ranges from 18-44 years, with a median age of 26 years and mean of 28.0 years (SD=7.6; mean and median data not presented). The sample was evenly split between females and males (50.1% versus 49.9%). A third of participants worked in the informal sector (e.g., craftsperson,

For peer review only

restauranteur; 34.9%); another third were currently students (31.8%). Most participants completed the survey via phone rather than online, respectively 84.1% and 15.9%.

We identified some differences between our two recruitment methods – i.e., online versus telephone (data not presented). Compared to participants recruited online, participants recruited telephonically were more likely to be older on average (telephone=28.5 years versus online=25.0; $p<.001$), live in rural versus urban settings (telephone=23.0% rural versus online=4.4%; $p<.001$), and have less than college education (telephone=16.1% college-educated versus online=79.7%; $p<.001$). Half of telephone participants were male, compared to less than four in ten online participants (49.9% telephone versus 39.4% online; $p=.003$). Significant differences were also identified between recruitment methods for relationship status ($p<.001$), profession ($p<.001$), and religion ($p<.001$). Compared to telephone participants, online participants also had significantly higher COVID-19 knowledge and awareness ($p<.001$) and were significantly more likely to engage in prevention behaviors ($p<.001$).

RQ1: In Senegal, what is the level of knowledge and awareness about COVID-19?

As shown in Table 2, of the 1,452 participants in our study sample less than 1% had not heard about COVID-19 (0.4%). More than nine in ten participants knew that hand washing (94.8%) and mask wearing in public (97.8%) were effective methods to prevent COVID-19 transmission and/or infection. About half were aware that staying at home (48.6%) or avoiding groups of more than 10 people (50.8%) were COVID-19 prevention recommendations. Nearly four in five participants (79.4%) said that they would know who to contact (e.g., testing, healthcare) if they suspected themselves to

For peer review only - http://bmjopen.bmj.com/site/about/guidelines.xhtml

have had COVID-19. The median knowledge score was 12.5 out of 15 (range=0 [lowest] to 15 [highest]; mean=11.8 / SD=2.6; data not presented).

RQ2: To what extent is COVID-19 perceived as a health threat?

As shown in Table 2, nine in ten participants (94.6%) perceived COVID-19 to be a very or somewhat serious danger to their health. Almost a quarter (73.3%) said that COVID-19 posed a serious risk to Africans. Median perceived threat score was 11.6 out of 15 (mean=10.9 /SD=3.5; data not presented).

RQ3: What is the relationship between COVID-19 beliefs and prevention behaviors?

As shown in Table 2, nine in ten participants reported washing their hands multiple times per day (94.4%). Nearly eight in ten used hand sanitizer multiple times per day (77.4%). Approximately seven in ten participants wore masks every time they left their home (65.6%). Six in ten reported practicing social distancing all or most of the time (59.3%). Half of participants reported staying home all or most of the time (52.3%). Less than one in ten people went out as usual (6.2%) or ignored social distancing (5.7%). Less than one in one hundred participants never wore a mask in public (0.4%). The median prevention behaviors score was 11 out of 15 (mean=10.5/ SD=2.3; data not presented).

Regression Analysis As shown in Figure 1 and Table 3, COVID-19 behaviors were significantly associated with COVID-19 beliefs and participant demographic attributes. We also observed differences in behaviors based on our participant recruitment method. Compared to participants recruited telephonically, participants

For peer review only - http://bmjopen.bmj.com/site/about/guidelines.xhtml

recruited online were significantly more likely to socially distance (OR=3.33; p=<.001), wear masks (OR=3.26; p=.007), and stay home (OR=1.63; p=.013).

Participants who reported high degrees of perceived threat about COVID-19 were significantly more likely to practice prevention behaviors in both the aggregate and individual models (OR=1.69; p<.001). Knowledge and awareness related to COVID-19 poorly predicted practicing prevention behaviors, although high levels of knowledge and awareness were positively associated with participants' use of hand sanitizer (OR=1.47; p=.035). At the same time, higher levels of knowledge and awareness about COVID-19 was negatively associated with staying home (OR=0.64; *p*=.002).

Compared to participants with less than a high school degree, participants with high school education or above were more likely to stay home (OR=1.67; p<.001), practice social distancing (OR=1.28; p=.070), and use hand sanitizer (OR=1.35; p=.062). Compared to urban participants, rural participants were more likely to socially distance (OR=1.36; p=.032), stay home (OR=1.80; p<.001), and wear a mask (OR=2.17; p=.021), but less likely to user hand sanitizer (OR=0.67; p=.010). Use of hand sanitizer was more likely among formal workers compared to students (OR=2.18; p=.002). Compared to participants less than 26 years old, older participants (over 26) were significantly less likely to stay home (OR=0.76; *p*=.045).

Gender-based differences were observed for COVID-19 prevention behaviors. Men had 41% lower odds of practicing *all five* COVID-19 prevention behaviors compared to women (OR=0.59; p<.001). Men also reported lower adherence to COVID-19 prevention measures for each of the five behaviors individually. Compared to women, men were 61% less likely to wash their hands on a daily basis (p<.001), 51%

For peer review only - http://bmjopen.bmj.com/site/about/guidelines.xhtml

less likely to report staying home (p<.001), 39% less likely to wear masks daily (p<.037), 38% less likely to practice social distancing (p<.001), and 37% less likely to use hand sanitizer (p=.001).

For peer review only

Table 1. Sample characteristics. N=1,452. Data collected via phone and online surveys from Senegalese adults between June-August 2020.

Characteristic	Category	#	%
Survey modality	Telephone	1,221	84.1
	Online	231	15.9
Age Quartile	<21	353	24.3
	22-25	352	24.2
	26-34	354	24.4
	35+	393	27.1
Sex	Female	752	51.8
	Male	700	48.2
Location	Urban	1,118	77.1
	Rural	291	20.1
	Urban + Rural	42	2.9
Education	Superior	381	26.2
	Secondary	344	23.7
	Primary	284	19.6
	Middle	231	15.9
	No	145	10.0
	Other	67	4.6
Marital status	Single	804	55.4
	Married - monogamous	460	31.7
	Married - polygamist	161	11.1
	Divorced	20	1.4
	Widower	7	0.5
Occupation	Informal sector	569	39.2
	Student	400	27.6
	Formal sector	225	15.5
	Other	112	7.7
	Housewife	96	6.6
	Retired, out of work	49	3.4
	Religious leader	1	0.1
Religion	Muslim	1,372	94.5
	Catholic	65	4.5
	Protestant / Evangelical	8	0.6
	No	3	0.2
	Other	3	0.2
	Traditionalist	1	0.1

For peer review only

Table 2. Frequency (#) and percent (%) for responses to COVID-19 questionnaire items. N=1,452 Senegalese adult participants. All responses collected between June-August 2020.

	#	%
**Knowledge and Awareness Items**		
Have you ever heard of COVID-19, also called Coronavirus?		
Yes	1,446	99.6
No	6	0.4
What protective measure against COVID-19 / coronavirus do you know?		
Stay at home	705	48.6
Wash your hands regularly	1,377	94.8
Wear a mask in public	1,420	97.8
Avoid meeting with groups of more than 10 people	738	50.8
**Prevention Behavior Items (Past Week)**		
Staying at home		
Partly (I try to limit time away from home but go out several times a week)	602	41.5
Most of the time (I go out about once a week)	476	32.8
All the time (I didn't leave the house)	282	19.5
I went out as much as usual	90	6.2
Wash your hands with soap and water		
Several times a day, even if they do not seem dirty	830	57.4
Every day, as soon as they are dirty after the toilet, the kitchen or the work	535	37.0
Very rarely / Never	44	3.0
Once a day approximately	38	2.6
Wear a mask when you leave your home		
Each time	951	65.6
Most of the time	403	27.8
Sometimes	90	6.2
Never / I don't have a mask	6	0.4
Use hand sanitizer gel		
Every day, but only in the places where it is needed	595	41.0
Several times a day	528	36.4
Rarely / never	215	14.8
About once a day	112	7.7
Practice social distancing (limit the number of other people)		
Most of the time (I go out rarely and keep a distance)	557	38.4
Partly (I don't limit my social interactions but I keep my distance)	507	35.0
All the time (I am confined to my home)	303	20.9
Never (I act as usual: I pass other people and I shake their hands)	83	5.7
**Perceived Threat**		
Do you think COVID-19 represents a danger to your health?		
Yes, very serious	1,152	79.6
Somewhat serious	217	15.0

For peer review only

No	78	5.4
Are you afraid of catching COVID-19?		
Very afraid	913	63.1
Relatively afraid	347	24.0
Not afraid	186	12.9
Do you think the COVID-19 epidemic poses a risk to Africans?		
Yes, a serious risk	1,061	73.3
Yes, but a reasonable risk	283	19.5
No, a low risk	104	7.2
If you thought you had caught COVID-19, would you know who to contact?		
Yes	1,149	79.4
No	298	20.6
How stressed do you feel about hearing about COVID-19?		
Somewhat stressed	586	40.5
Stressed a little	366	25.3
Stressed a lot	262	18.1
Not at all stressed	234	16.2

For peer review only

Table 3. Logistic regression models predicting COVID-19 prevention behaviors (aggregate + individually). N=1,452. All estimates presented are adjusted for other model variables. All responses collected between June-August 2020.

	**All Behaviors**				**Using Hand Sanitizer**				**Social Distancing**			
Predictor	*OR*	*95% CI*		*p*	*OR*	*95% CI*		*p*	*OR*	*95% CI*		*p*
Online participant	1.93	1.29	2.87	0.001	1.62	0.94	2.78	0.079	3.20	2.05	5.00	<.001
COVID Perceived threat	1.69	1.33	2.13	<.001	1.69	1.28	2.22	<.001	2.03	1.61	2.57	<.001
Rural	1.49	1.12	1.98	0.006	0.67	0.50	0.91	0.010	1.36	1.03	1.81	0.032
High school or above	1.33	1.01	1.76	0.044	1.35	0.98	1.84	0.062	1.28	0.98	1.67	0.070
Formal worker	1.28	0.86	1.90	0.230	2.18	1.32	3.60	0.002	1.42	0.95	2.13	0.090
Informal worker	1.07	0.76	1.52	0.691	1.33	0.90	1.97	0.149	0.98	0.70	1.38	0.927
COVID KAP	0.96	0.72	1.29	0.798	1.47	1.03	2.10	0.035	0.89	0.66	1.18	0.411
Single	0.75	0.56	1.02	0.070	1.20	0.86	1.69	0.281	0.79	0.59	1.06	0.116
26 years or older	0.75	0.56	1.00	0.054	1.19	0.86	1.64	0.287	0.78	0.59	1.03	0.077
Male	0.59	0.46	0.75	<.001	0.64	0.49	0.84	0.001	0.62	0.49	0.79	<.001

	**Staying Home**				**Hand Washing**				**Mask Wearing**			
Predictor	*OR*	*95% CI*		*p*	*OR*	*95% CI*		*p*	*OR*	*95% CI*		*p*
Online participant	1.62	1.09	2.42	0.018	2.48	0.89	6.96	0.084	3.25	1.31	8.04	0.011
COVID Perceived threat	1.62	1.29	2.03	<.001	3.09	1.67	5.71	<.001	2.81	1.64	4.82	<.001
Rural	1.80	1.35	2.38	<.001	1.52	0.81	2.86	0.189	2.17	1.12	4.19	0.021
High school or above	1.67	1.28	2.17	<.001	0.85	0.49	1.45	0.546	0.82	0.50	1.36	0.448
Formal worker	0.98	0.67	1.44	0.918	1.54	0.67	3.53	0.311	2.44	1.03	5.75	0.042
Informal worker	0.89	0.64	1.25	0.510	1.12	0.58	2.16	0.733	1.10	0.60	1.99	0.760
COVID KAP	0.64	0.48	0.85	0.002	1.78	0.88	3.63	0.110	1.14	0.64	2.04	0.654
Single	0.85	0.64	1.13	0.260	0.93	0.50	1.71	0.809	0.73	0.41	1.29	0.277
26 years or older	0.76	0.58	0.99	0.045	1.40	0.80	2.46	0.243	1.05	0.63	1.77	0.847
Male	0.49	0.39	0.62	<.001	0.39	0.23	0.66	<.001	0.61	0.39	0.97	0.037

For peer review only - http://bmjopen.bmj.com/site/about/guidelines.xhtml

Discussion

In our cross-sectional sample of Senegalese adults, we observed that the majority of participants practiced COVID-19 prevention behaviors on a daily basis. Mask wearing, hand washing, and use of hand sanitizer were most frequently reported. Social distancing and staying at home were also reported albeit to a lower degree. We also identified a range of psychosocial and demographic predictors for COVID-19 prevention behaviors. For example, men and women differed in the degree to which they practiced COVID-19 prevention behaviors. Participants who perceived COVID-19 to be a threat were significantly more likely to practice every prevention behavior measured than those who did not perceive COVID-19 to be a threat. This is in line with existing health behavior research regarding perceived threat as a necessary precursor to behavior change.[17,18]

Prevention Behaviors

Men were less likely than women to report practicing any of the prevention behaviors that we studied. Other studies have shown that women are more likely to engage in preventive behaviors,[23,24] including towards COVID-19.[25,26] Men may also be more likely to engage in risk taking behavior,[27] whereas women are more risk averse.[28] Practicing COVID-19 prevention behaviors was also positively associated with living in a rural vs. urban area. It is possible that people residing in rural areas have an easier time implementing preventive behaviors such as staying at home and social distancing than people in more densely populated urban areas. Participants with at least a high school education were also more likely to engage in preventive behaviors. Educational differences in adoption of preventive health behaviors has previously been reported.[23,29] Demographic variations in practicing COVID-19 prevention behaviors indicate priority populations for future health communication

For peer review only

campaigns. For example, people in urban areas may require more prompting and resources to implement preventive behaviors in the future.

Our results suggest that online communication will be an important strategy to build vaccine confidence and to continue supporting prevention behaviors especially as vaccine availability increases throughout the region. Respondents from the online sample demonstrated greater knowledge and awareness of COVID-19 and engaged in prevention behaviors more often compared to respondents in the telephone sample. This provides support for both online and on-the-ground messaging and communication to strengthen pro-prevention beliefs, attitudes, and behaviors.

Health Beliefs

Our study findings comport with a robust body of evidence about COVID-19 knowledge, awareness, attitudes, and practices. In Senegal, we observed that knowledge, perceived risk, and practicing prevention behaviors for COVID-19 were very high in general – albeit with some demographic variations. A 2020 international review by Puspitasari and colleagues reported generally high knowledge and awareness about COVID-19, pro-prevention attitudes, and high adherence to COVID-19 prevention behaviors like handwashing and social distancing.[30] Closer to the West African and Senegalese contexts, Aduh and colleagues (2020) reported that in sub-Saharan Africa moderate to high levels of COVID-19 knowledge and perceived risk.[15]

Contrary to prior international research, we did not find an association between knowledge and engaging in prevention behaviors. Rather, perceived COVID-19 risk was a stronger predictor of prevention behaviors, which is also supported by previous international research.[2,31] Low perceptions of risk may result in low compliance with virus restrictions and

For peer review only

adherence to prevention behaviors,[32–34] and therefore facilitates COVID-19's spread. This confirms another recent study which recruited using social media from the United States, China, Taiwan, and Mexico.[2] Hsing and colleagues found that perceived COVID-19 severity and susceptibility – constructs that comprise perceived risk/threat in the Health Belief Model – were both predictors of hand washing and social distancing.[2] In our study based in Senegal, perceived risk not only predicted hand washing and social distancing, it also predicted staying home, using hand sanitizer, and mask wearing. These findings suggest that to prevent COVID-19 spread in Senegal and other parts of francophone West Africa, we need to focus more on perceived risk than knowledge.

A Focus on Senegal

Senegal's communication regarding COVID-19 has widely been considered successful.[35,36] In general, Senegalese participants had high levels of COVID-19 knowledge and awareness, perceived COVID-19 to be a threat both personally and across Senegal, and in most cases adopted known prevention behaviors on a daily or regular basis. Looking forward, as vaccines become more widely available in Senegal and many West African nations, inequities in access will make sustaining preventive behaviors paramount to continued control of the virus.[37] Our findings suggest that other nations should look to Senegalese communications about COVID-19 to learn messages that may resonate more effectively with their populations to increase prevention behaviors.

As has been stated, most participants reported moderate to high adherence to COVID-19 prevention behaviors. To explain our findings, we must consider lessons learned from previous regional epidemics and how they relate to evolving social contexts (e.g., norms, culture, population attributes).[20] COVID-19 in West Africa comes several years after the

For peer review only - http://bmjopen.bmj.com/site/about/guidelines.xhtml

Ebola outbreak in 2014-2015, which tested the limits of the health systems. For example, although Senegal experiences a nationwide shortage of doctors – just 7 per 100,000 persons in 2020 – the nation has been comparatively effective at controlling COVID-19 infections. The first case of COVID-19 in Senegal, a country of 17 million, was reported in early March 2020, prompting the Ministry of Health to develop protocols to monitor and address cases within Senegal and at its borders and airports. Cases in Senegal remained low throughout Spring and Summer 2020, but like other nations rose considerably in the first months of 2021.[38,39]

Senegal's success at controlling the virus throughout 2020 has been attributed to many factors, including a citizenry who has witnessed the devastating impacts of the Ebola epidemic. More broadly across West Africa, citizens may be more likely to comply with public health restrictions because of their experience with Ebola,[40] and this extends to individual behavior changes as well. For example, one study reported that handwashing was shown to increase in Senegal as a result of Ebola education campaigns.[41] In our analysis, we found that daily hand washing was the prevention behavior most often reported by participants. Future health campaigns may look at Ebola prevention messages to identify behaviors and information that may be reinforced in the context of COVID-19.

Limitations

The current study is not without limitations. First, our findings may not be generalizable beyond Senegal, or more specifically Senegalese adults. Second, our recruitment strategy increased the potential for selection bias because participants were recruited online and on-the-ground in Senegal; thus, all participants, by the nature of the recruitment methods, had access to the internet and/or a cell phone. Selection bias is common in online and social media recruitment because recruited participants tend to be more well-educated and younger than

For peer review only

the general population.[2,42] To address potential confounding between recruitment methods, we controlled for recruitment method in our multivariate regression modelling. At the same time, we believe that our multi-modal recruitment strategy bolsters the representativeness of our sample and is a key strength of our study. Had we excluded online participants, then our sample's demographic characteristics would likely have differed. Conversely, had telephone participants been excluded then our sample would have skewed older, female, and educated – social media sample biases that we addressed through dual data collection methods.

Third and finally, we did not identify significant relationships for many factors in our regression model because our study was not statistically powered to detect significant differences. For example, COVID-19 knowledge was high in our sample and an insignificant predictor of prevention behaviors. A study with a larger sample may have been able to identify relationships between knowledge and behaviors. However, our study adapted pre-existing research panels and developed novel data collection instruments to capture information about COVID-19, and this may serve as a model for the responsiveness of ongoing scholarship to future global events.

Conclusion

In the context of COVID-19, researchers must learn to apply traditional research methodologies online, such as on social media, to shed light on the etiology of COVID-19 in vulnerable and marginalized populations (Ali et al., 2020; Bavel et al., 2020). Such work creates evidence that may not only be applied to other places in developing regions but also enhance representation in research, which is crucial to creating tailored and effective interventions (Redwood & Gill, 2013). More broadly, research that creates evidence and informs policy about health issues like COVID-19 in West Africa and other parts of the

For peer review only - http://bmjopen.bmj.com/site/about/guidelines.xhtml

developing world promotes global health equity and justice (Braveman & Gruskin, 2003; Cacari-Stone et al., 2014; Östlin et al., 2011).

Our study contributes to a growing body of literature about COVID-19 in Africa,[43,44] and is the first study to look specifically at knowledge and prevention behaviors in a francophone West African nation. In Senegal, we observed that knowledge and perceived risk of COVID-19 were very high in general. But, risk was a stronger and more influential predictor of COVID-19 prevention behaviors. COVID-19 prevention behaviors also varied by gender, education, location, and country. These findings suggest that in West Africa, nations should seek to elevate risk perceptions about COVID-19 and develop messaging to better target certain groups. Elevating the perceived risk of a population is a difficult issue because public health programs do not want to raise unnecessary alarm. Thus, messages around risk of COVID-19 should be accompanied by empowering messages of how individuals can protect themselves, their families, and their communities. Health campaigns should look backwards to successes with Ebola for improving health messaging about COVID-19, and may also look to replicate the successes of regional examples like Senegal.

For peer review only

Data availability statement

No data are available.

Ethics statements

Patient consent for publication: Not required.

Research ethics approval: All study procedures and protocols were approved by Drexel University's institutional ethical review board (Protocol ID: 00015164). Written informed consent was obtained from all participants.

Acknowledgements

We are grateful for support from our French and Senegalese collaborators in Dakar, Senegal at The African Health and Education Network (NGO RAES) and at Université Cheikh-Anta-Diop. Data collection was completed while the first (MK) and last author (PM) were affiliated with the Drexel University Dornsife School of Public Health, and while the second author (MB) was a graduate student at UCLA Fielding School of Public Health. Finally, we are thankful to all participants in this study.

Competing Interests

None declared.

Funding

This study received funding from the Bill & Melinda Gates Foundation (OPP1181104). Disclaimer. The study sponsors had no role in study design, data collection, data analysis, our decision to publish, or preparing the manuscript.

Contributors

All authors contributed to the editing and reviewing of this paper. MK, MB, DG, and PM were involved in conceptualizing this article. MF and RN led development of the data collection tool. MK and RN designed analyses for this manuscript, and MK conducted the analyses. MK, MB, DG, and PM wrote the paper. All authors reviewed, edited, and revised the paper.

For peer review only

References

1 Naidoo AV, Hodkinson P, Lai King L, *et al.* African authorship on African papers during the COVID-19 pandemic. *BMJ Glob Health* 2021;**6**:e004612. doi:10.1136/bmjgh-2020-004612

2 Hsing JC, Ma J, Barrero-Castillero A, *et al.* Influence of Health Beliefs on Adherence to COVID-19 Preventative Practices: International, Social Media–Based Survey Study. *J Med Internet Res* 2021;**23**:e23720. doi:10.2196/23720

3 Alistair Mallard, Maria Amalia Pesantes, Carol Zavaleta-Cortijo, *et al.* An urgent call to collect data related to COVID-19 and Indigenous populations globally. *BMJ Glob Health* 2021;**6**:e004655. doi:10.1136/bmjgh-2020-004655

4 Sarah Wehbe, Fahme SA, Rizk A, *et al.* COVID-19 in the Middle East and North Africa region: an urgent call for reliable, disaggregated and openly shared data. *BMJ Glob Health* 2021;**6**:e005175. doi:10.1136/bmjgh-2021-005175

5 Connolly KK, Crosby ME. Examining e-Health Literacy and the Digital Divide in an Underserved Population in Hawai'i. *Hawaii J Med Public Health* 2014;**73**:44–8.

6 Lavery MPJ, Abadi MM, Bauer R, *et al.* Tackling Africa's digital divide. *Nat Photonics* 2018;**12**:249–52. doi:10.1038/s41566-018-0162-z

7 Lopez MH, Gonzalez-Barrera A, Patten E. Closing the Digital Divide: Latinos and Technology Adoption. Pew Hisp. Cent. 2013.

8 Bodewes AJ, Kunst AE. Involving hard-to-reach ethnic minorities in low-budget health research: lessons from a health survey among Moluccans in the Netherlands. *BMC Res Notes* 2016;**9**:319. doi:10.1186/s13104-016-2124-1

9 Dusek G, Yurova Y, P. Ruppel C. Using Social Media and Targeted Snowball Sampling to Survey a Hard-to-reach Population: A Case Study. *Int J Dr Stud* 2015;**10**:279–99. doi:10.28945/2296

10 Wilkerson JM, Iantaffi A, Grey JA, *et al.* Recommendations for Internet-Based Qualitative Health Research With Hard-to-Reach Populations. *Qual Health Res* 2014;**24**:561–74. doi:10.1177/1049732314524635

11 Gifford P. Religion in Contemporary Senegal. *J Contemp Relig* 2016;**31**:255–67. doi:10.1080/13537903.2016.1152684

12 UNDP, editor. *The next frontier: human development and the Anthropocene*. New York, NY: : United Nations Development Programme 2020.

13 Chitungo I, Dzobo M, Hlongwa M, *et al.* COVID-19: Unpacking the low number of cases in Africa. *Public Health Pract* 2020;**1**:100038. doi:10.1016/j.puhip.2020.100038

For peer review only

14 Mbow M, Lell B, Jochems SP, *et al.* COVID-19 in Africa: Dampening the storm? *Science* 2020;**369**:624–6. doi:10.1126/science.abd3902

15 Aduh U, Folayan MO, Afe A, *et al.* Risk perception, public health interventions, and Covid-19 pandemic control in sub-saharan Africa. *J Public Health Afr* Published Online First: 9 October 2020. doi:10.4081/jphia.2021.1622

16 Worldometer. Realtime Tracker: Population of Western Africa. 2021.https://www.worldometers.info/world-population/western-africa-population/ (accessed 22 Apr 2021).

17 Glanz K, Rimer BK, Viswanath K. *Health Behavior: Theory, Research, and Practice*. John Wiley & Sons 2015.

18 Rosenstock IM. Historical Origins of the Health Belief Model. *Health Educ Monogr* 1974;**2**:328–35. doi:10.1177/109019817400200403

19 Abbasi-Kangevari M, Kolahi A-A, Ghamari S-H, *et al.* Public Knowledge, Attitudes, and Practices Related to COVID-19 in Iran: Questionnaire Study. *JMIR Public Health Surveill* 2021;**7**:e21415. doi:10.2196/21415

20 Bavel JJV, Baicker K, Boggio PS, *et al.* Using social and behavioural science to support COVID-19 pandemic response. *Nat Hum Behav* 2020;**4**:460–71. doi:10.1038/s41562-020-0884-z

21 Harper CA, Satchell LP, Fido D, *et al.* Functional Fear Predicts Public Health Compliance in the COVID-19 Pandemic. *Int J Ment Health Addict* Published Online First: 27 April 2020. doi:10.1007/s11469-020-00281-5

22 Gordon M, Lumley T, Gordon MM. Package ‘forestplot’. *Adv For Plot Using ‘grid’Graphics* 2016.

23 Berrigan D, Dodd K, Troiano RP, *et al.* Patterns of health behavior in U.S. adults. *Prev Med* 2003;**36**:615–23. doi:10.1016/S0091-7435(02)00067-1

24 Liang W, Shediac-Rizkallah MC, Celentano DD, *et al.* A population-based study of age and gender differences in patterns of health-related behaviors. *Am J Prev Med* 1999;**17**:8–17. doi:10.1016/S0749-3797(99)00040-9

25 Bronfman N, Repetto P, Cordón P, *et al.* Gender Differences on Psychosocial Factors Affecting COVID-19 Preventive Behaviors. *Sustainability* 2021;**13**:6148. doi:10.3390/su13116148

26 Yıldırım M, Geçer E, Akgül Ö. The impacts of vulnerability, perceived risk, and fear on preventive behaviours against COVID-19. *Psychol Health Med* 2021;**26**:35–43. doi:10.1080/13548506.2020.1776891

For peer review only

27 Byrnes JP, Miller DC, Schafer WD. Gender differences in risk taking: A meta-analysis. *Psychol Bull* 1999;**125**:367–83. doi:10.1037/0033-2909.125.3.367

28 Adhikari B, O'Leary VE. Gender Differences in Risk Aversion: A Developing Nation's Case. Rochester, NY: : Social Science Research Network 2011. https://papers.ssrn.com/abstract=1522974 (accessed 9 Sep 2021).

29 Chen X, Chen H. Differences in Preventive Behaviors of COVID-19 between Urban and Rural Residents: Lessons Learned from A Cross-Sectional Study in China. *Int J Environ Res Public Health* 2020;**17**:4437. doi:10.3390/ijerph17124437

30 Puspitasari IM, Yusuf L, Sinuraya RK, *et al.* Knowledge, Attitude, and Practice During the COVID-19 Pandemic: A Review. J. Multidiscip. Healthc. 2020;**13**:727–33. doi:10.2147/JMDH.S265527

31 Li S, Feng B, Liao W, *et al.* Internet Use, Risk Awareness, and Demographic Characteristics Associated With Engagement in Preventive Behaviors and Testing: Cross-Sectional Survey on COVID-19 in the United States. *J Med Internet Res* 2020;**22**:e19782. doi:10.2196/19782

32 Chertok IRA. Perceived risk of infection and smoking behavior change during COVID-19 in Ohio. *Public Health Nurs* 2020;**n/a**. doi:10.1111/phn.12814

33 Gierlach E, Belsher BE, Beutler LE. Cross-cultural differences in risk perceptions of disasters. *Risk Anal Off Publ Soc Risk Anal* 2010;**30**:1539–49. doi:10.1111/j.1539-6924.2010.01451.x

34 Slovic P, Fischhoff B, Lichtenstein S. Why Study Risk Perception? *Risk Anal* 1982;**2**:83–93. doi:10.1111/j.1539-6924.1982.tb01369.x

35 BBC News. Coronavirus in Senegal: Keeping Covid-19 at bay. BBC News. 2020.https://www.bbc.com/news/world-africa-54388340 (accessed 30 Mar 2021).

36 FP Analytics. The COVID-19 Global Response Index: A country-by-country assessment of government responses to the pandemic. Foreign Policy. 2021.https://globalresponseindex.foreignpolicy.com/ (accessed 30 Mar 2021).

37 Reuters. 'Immense joy' as Senegal begins public vaccine distribution. Reuters. 2021.https://www.reuters.com/article/us-health-coronavirus-senegal-vaccinatio-idUSKBN2AO2OH (accessed 30 Mar 2021).

38 Dia N, Lakh NA, Diagne MM, *et al.* COVID-19 Outbreak, Senegal, 2020. *Emerg Infect Dis* 2020;**26**:2771–3. doi:10.3201/eid2611.202615

39 Dong E, Du H, Gardner L. An interactive web-based dashboard to track COVID-19 in real time. *Lancet Infect Dis* 2020;**20**:533–4. doi:10.1016/S1473-3099(20)30120-1

For peer review only

40 Rosenthal PJ, Breman JG, Djimde AA, *et al.* COVID-19: Shining the Light on Africa. *Am J Trop Med Hyg* Published Online First: 5 May 2020. doi:10.4269/ajtmh.20-0380

41 Sougou NM, Diouf J, Leye MMM, *et al.* What community-based public health approaches in West Africa for COVID-19 epidemic? A reflection based on the African socio-cultural context. *Pan Afr Med J* 2020;**35**. doi:10.11604/pamj.supp.2020.35.2.24415

42 Pew Research Center. Demographics of Social Media Users and Adoption in the United States. Pew Res. Cent. Internet Sci. Tech. 2019.https://www.pewresearch.org/internet/fact-sheet/social-media/ (accessed 31 Mar 2021).

43 Gesesew HA, Koye DN, Fetene DM, *et al.* Risk factors for COVID-19 infection, disease severity and related deaths in Africa: a systematic review. *BMJ Open* 2021;**11**:e044618. doi:10.1136/bmjopen-2020-044618

44 Kana MA, LaPorte R, Jaye A. Africa's contribution to the science of the COVID-19/SARS-CoV-2 pandemic. *BMJ Glob Health* 2021;**6**:e004059. doi:10.1136/bmjgh-2020-004059

For peer review only

Figure 1. Forest plot diagrams for logistic regression models predicting COVID-19 prevention behaviors (aggregate + individually). Square box represents point estimate and horizontal bar represents 95% CI. Confidence intervals that cross OR threshold of 1.0 are not statistically significant at the alpha=0.05 level. All estimates presented are adjusted for other model variables. N=1,452. All responses collected between June-August 2020.

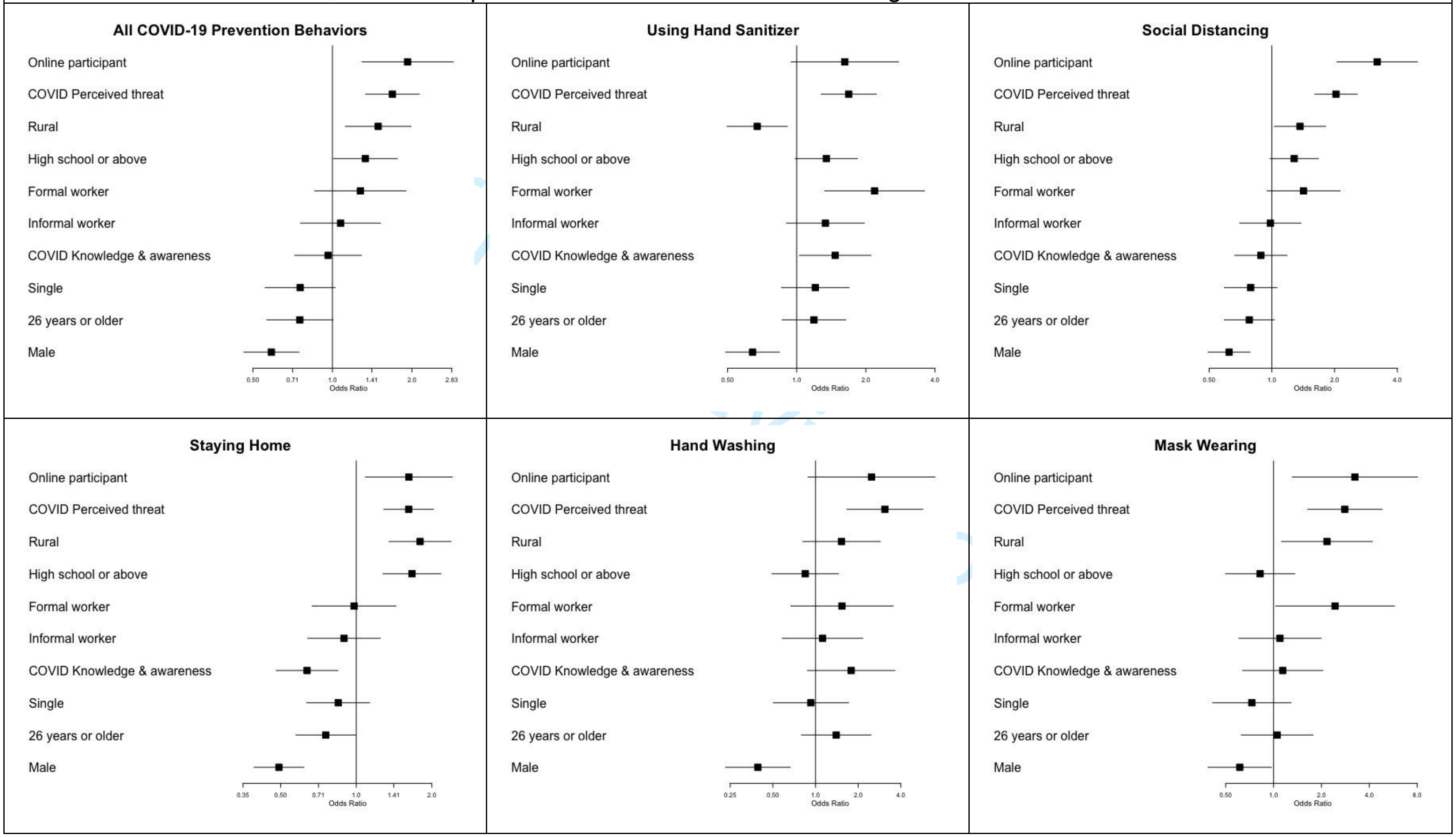

BMJ Open

A cross-sectional study of COVID-19 knowledge, beliefs and prevention behaviors among adults in Senegal

Journal:	*BMJ Open*
Manuscript ID	bmjopen-2021-057914.R1
Article Type:	Original research
Date Submitted by the Author:	01-Feb-2022
Complete List of Authors:	Kearney, Matthew; University of Pennsylvania Perelman School of Medicine, Family Medicine and Community Health<br>Bornstein, Marta; The Ohio State University<br>Fall, Marieme; The African Health and Education Network (NGO RAES)<br>Nianogo, Roch; UCLA School of Public Health, Epidemiology<br>Glik, D; UCLA, Community Health Sciences<br>Massey, Philip; University of Arkansas Fayetteville, Health, Human Performance, and Recreation
**Primary Subject Heading**:	Public health
Secondary Subject Heading:	Global health, Infectious diseases
Keywords:	COVID-19, Public health < INFECTIOUS DISEASES, PUBLIC HEALTH

SCHOLARONE™
Manuscripts

I, the Submitting Author has the right to grant and does grant on behalf of all authors of the Work (as defined in the below author licence), an exclusive licence and/or a non-exclusive licence for contributions from authors who are: i) UK Crown employees; ii) where BMJ has agreed a CC-BY licence shall apply, and/or iii) in accordance with the terms applicable for US Federal Government officers or employees acting as part of their official duties; on a worldwide, perpetual, irrevocable, royalty-free basis to BMJ Publishing Group Ltd ("BMJ") its licensees and where the relevant Journal is co-owned by BMJ to the co-owners of the Journal, to publish the Work in this journal and any other BMJ products and to exploit all rights, as set out in our licence.

The Submitting Author accepts and understands that any supply made under these terms is made by BMJ to the Submitting Author unless you are acting as an employee on behalf of your employer or a postgraduate student of an affiliated institution which is paying any applicable article publishing charge ("APC") for Open Access articles. Where the Submitting Author wishes to make the Work available on an Open Access basis (and intends to pay the relevant APC), the terms of reuse of such Open Access shall be governed by a Creative Commons licence – details of these licences and which Creative Commons licence will apply to this Work are set out in our licence referred to above.

Other than as permitted in any relevant BMJ Author's Self Archiving Policies, I confirm this Work has not been accepted for publication elsewhere, is not being considered for publication elsewhere and does not duplicate material already published. I confirm all authors consent to publication of this Work and authorise the granting of this licence.

For peer review only

Title

A cross-sectional study of COVID-19 knowledge, beliefs and prevention behaviors among adults in Senegal

Authors

Matthew D. Kearney PhD MPH – Department of Family Medicine and Community Health, Perelman School of Medicine, University of Pennsylvania, Philadelphia, Pennsylvania, USA, ORCID ID: 0000-0003-2898-573X

Marta Bornstein PhD MPH – College of Public Health, The Ohio State University, Columbus, OH, USA, ORCID ID: 0000-0002-5200-8181

Marieme Fall MPH – African Health and Education Network (RAES), Dakar, Senegal

Roch Nianogo PhD – Department of Epidemiology, UCLA Fielding School of Public Health, Los Angeles, CA, USA, ORCID ID: 0000-0001-5932-6169

Deborah Glik PhD – Department of Community Health Sciences, UCLA Fielding School of Public Health, Los Angeles, CA, USA, ORCID ID: 0000-0002-2532-3661

Philip M. Massey PhD MPH – Department of Health, Human Performance, and Recreation, College of Education and Health Professions, University of Arkansas, Fayetteville, AR, USA, ORCID: 0000-0002-0577-8618

Corresponding Author

Name: Dr. Matthew D. Kearney

Address: Penn Presbyterian Medical Center, Andrew Mutch Building, 51 N. 39th Street, Philadelphia, PA 19104

Email: kearnm@pennmedicine.upenn.edu

Word Count: 4,660

For peer review only

Abstract

Objectives: The aim of the study was to explore COVID-19 beliefs and prevention behaviors in a francophone West African nation, Senegal.

Design: This was a cross-sectional analysis of survey data collected via a multi-modal observational study.

Participants: Senegalese adults aged 18 years or older (n=1,452).

Primary and secondary outcome measures: Primary outcome measures were COVID-19 prevention behaviors. Secondary outcome measures included COVID-19 knowledge and beliefs. Univariate, bivariate, and multivariate statistics were generated to describe the sample and explore potential correlations.

Setting: Participants from Senegal were recruited online and telephonically between June-August 2020.

Results: Mask wearing, hand washing, and use of hand sanitizer were most frequently reported. Social distancing and staying at home were also reported albeit to a lower degree. We also identified a range of psychosocial and demographic predictors for COVID-19 prevention behaviors. Men, compared to women, had lower odds (AOR=0.59, 95% CI: 0.46-0.75, p<.001) of reporting prevention behaviors. Rural residents (vs. urban; AOR=1.49, 95% CI: 1.12-1.98, p=.001) and participants with at least a high school education (vs. less than high school education; AOR=1.33, 95% CI: 1.01-1.76, p=.006) were more likely to report COVID-19 prevention behaviors.

Conclusions: In Senegal, we observed that knowledge and perceived risk of COVID-19 were very high in general. But, risk was a stronger and more influential predictor of

For peer review only

COVID-19 prevention behaviors. Stakeholders and decision makers in Senegal and across Africa can use place-based evidence like ours to address COVID-19 risk factors and intervene effectively with policies and programming. Use of both phone and online surveys enhances representation and study generalizability and should be considered in future research with hard-to-reach populations.

Article Summary

Strengths and limitations of this study

- The main strength of our study was the use of a multi-modal data collection strategy, online and via telephone, which bolstered the representativeness of our total sample within Senegal and is a key strength of our study. Had we relied on a single method, our sample's demographic characteristics would likely have differed while also introducing selection bias based on what type of participant had access to a phone versus the internet.
- Prior to our study, little evidence was available about how populations in francophone West Africa have responded to COVID-19 in their daily lives. We adapted pre-existing research panels and developed novel data collection instruments to capture information about COVID-19, and this may serve as a model for the responsiveness of ongoing scholarship to future global events.
- Although multi-modal, our recruitment strategy may have increased the potential for selection bias because participants were recruited online and via telephone in Senegal but not on-the-ground; thus, all participants, by the nature of the recruitment methods, had access to the internet and/or a telephone. To address

For peer review only - http://bmjopen.bmj.com/site/about/guidelines.xhtml

potential confounding between recruitment methods, we controlled for recruitment method in our multivariate regression modelling.

- A study with a larger sample may have been able to identify relationships between knowledge and behaviors, and our study was not design with enough statistical power to detect significant differences.
- The findings of our study may not be generalizable beyond Senegal, or more specifically Senegalese adults.

For peer review only

Introduction

COVID-19 is an ongoing threat to global public health since its emergence at the end of 2019, yet little is known about how populations in low-resource settings have responded to COVID-19 in their daily lives. Although research is emerging about COVID-19 infection, strategies for prevention, and risk factors for severe disease and mortality, there is a dearth of literature about COVID-19 beliefs, specifically in the West Africa region. For example, between January and September of 2020, just 4.3% of research articles about COVID-19 were focused on Africa or a specific African nation.[1] Given that ongoing prevention strategies implemented at the country, community, and individual level will be needed to curb the COVID-19 pandemic, it is critical to study health beliefs and adherence to prevention behaviors within specific social and political contexts.[2]

Providing relevant and timely research is essential for guiding evidence-based outreach and interventions, especially during public health emergencies. It is particularly important to advance equity within public health, and the health of populations, by understanding the social contexts and health beliefs among indigenous, marginalized, and vulnerable populations around the globe.[3,4] Francophone West Africa, in particular, is underrepresented in global health research because of language barriers to publication and collaboration, leading to more research in English-speaking nations like Nigeria and Ghana.[5–7]

In the context of COVID-19, researchers must learn to apply traditional research methodologies online, such as on social media, to shed light on the etiology of COVID-19 in vulnerable and marginalized populations (Ali et al., 2020; Bavel et al., 2020). Such

For peer review only

work creates evidence that may not only be applied to other places in developing regions but also enhance representation in research, which is crucial to creating tailored and effective interventions (Redwood & Gill, 2013). More broadly, research that creates evidence and informs policy about health issues like COVID-19 in West Africa and other parts of the developing world promotes global health equity and justice (Braveman & Gruskin, 2003; Cacari-Stone et al., 2014; Östlin et al., 2011). The current study provides timely, critical, and actionable information about COVID-19 beliefs and prevention behaviors for stakeholders, policy makers, and public health programs to reduce the burden of COVID-19 in West Africa by reporting on findings among a sample of adults from the West African nation of Senegal.

Senegal as a Case Study of COVID-19 Beliefs and Behaviors in West Africa

Senegal is the former capital of French West Africa and home to over 16 million people, and therefore may serve as a relevant case study for exploring COVID-19 beliefs in West Africa. Furthermore, Senegal's communication regarding COVID-19 has widely been considered successful and may have contributed to unexpectedly fewer COVID-19 cases and deaths.[8,9]. Compared to the burden of COVID-19 in Europe, the Americas, the Middle East and South Asia, the burden of COVID-19 in Africa has been quite low. Relative to the rest of the world, Africa defied initial expectations by experiencing the lowest burden of reported COVID-19 cases and deaths.[10,11] Questions remain about why COVID-19 infections are reportedly low in Africa, in particular in West Africa where there have been 377 cases per 100,000 persons cumulatively. Although a recent analysis by Aduh and colleagues (2020) reported on perceptions and behaviors towards COVID-19 across Sub-Saharan Africa, we have

For peer review only

much to learn from populations in densely populated West Africa, a comparatively more urban Sub-Saharan region home to more than 400 million people.[13]

Some researchers have questioned the accuracy of African COVID-19 case numbers,[10] hypothesizing that the burden of disease could be much higher than reported. Uncertainty about the true trajectory of COVID-19 in Africa may leave all continents vulnerable to future outbreaks, particularly regions with low herd immunity due to lack of exposure and vaccine resource constraints. It is likely that places that have not yet needed to adopt strong prevention efforts because of reportedly low case numbers therefore may be especially vulnerable to future outbreaks. Thus, despite relatively low case numbers, it is still vital to investigate COVID-19 health beliefs and prevention behaviors in West Africa peoples to identify potential correlates and predictors of viral prevention behaviors. Our study aims to contribute evidence towards this gap in the literature by adapting pre-existing research panels and developing novel data collection instruments to capture information about COVID-19, and this may serve as a model for the responsiveness of ongoing scholarship to future global events.

Study Objectives

To explore COVID-19 beliefs and prevention behaviors in francophone West Africa, the current study analyzes cross-sectional survey data collected from a sample of adults in Senegal. The survey, which was administered both online and via telephone, included a module on COVID-19 behaviors and beliefs. We sought to identify correlates between respondent demographic attributes and COVID-19 beliefs and behaviors. Our investigation was theoretically informed from Rosenstock's Health Belief Model (HBM),[14] a behavior change framework for public health practice positing that

For peer review only - http://bmjopen.bmj.com/site/about/guidelines.xhtml

knowledge of a potential negative outcome and perceived threat of that outcome precede behavior change.[15] Other recent studies have similarly relied on HBM constructs to explore COVID-19. Specifically, this study applies HBM constructs to answer the following questions:

1) In Senegal, what is the level of knowledge and awareness about COVID-19?

2) To what extent is COVID-19 perceived as a health threat?

3) What is the relationship between COVID-19 beliefs and prevention behaviors?

Methods

Study Design, Participants and Setting

From June-August 2020, we conducted a multi-modal cross-sectional survey that included questions regarding COVID-19 beliefs and prevention behaviors among a convenience sample of 1,452 adults in Senegal. The survey was administered via the internet and telephone, which was appropriate given the constraints of research during COVID-19. Respondents were participating in a separate ongoing longitudinal cohort study.

Data Collection

This survey used two modes of data collection: online and telephone. We opted to collect data about COVID-19 from pre-existing panels of participants in Senegal so as to be responsive to the ongoing and evolving global epidemic and to address evidence gaps mentioned above (see Introduction). The telephone survey used a random selection from a panel of phone numbers of individuals who lived in Senegal and agreed to participate in media-related research. A quota system was used to match the overall

For peer review only

population of Senegal on sex and have an even distribution of age and level of education. A total of 1,221 participants were enrolled in the study via telephone, split approximately equally between participants living insider versus outside of Dakar, Senegal and by gender. Online participants from Senegal were recruited using social media advertisements on Facebook and YouTube. All online participants were part of a cohort study about entertainment education in West Africa that began in late summer 2019; of the 1,286 cohort participants recruited online in summer 2019, 231 both were eligible to participate (i.e., Senegalese adults) and completed the COVID-19 questionnaire items. Through our multi-modal strategy, responses about COVID-19 were collected from 1,452 Senegalese adults. Figure 1 presents a flow diagram of our study recruitment and sampling.

For both online and telephone participants, all individuals were required to be 18 years or older. Potential participants were informed about the study and provided consent to participate in the survey (online) or interview (telephone). Consenting participants were compensated with 2000 Central African CFA franc (~4 USD) for their time. Telephone surveys were collected by OMedia, a Dakar-based third party research firm, and overseen by researchers from UCLA (MB, DG). Online surveys were collected via Qualtrics by researchers from Drexel University (MK, PM). Incomplete responses with missing data were excluded. All study procedures and protocols were reviewed by and approved or determined to be exempt by institutional ethical review boards.

Measurement

Survey items included a series of self-reported demographic questions (e.g., age, education) as well as a 15-item section specifically about COVID-19. Using Cronbach's

For peer review only - http://bmjopen.bmj.com/site/about/guidelines.xhtml

α, we assessed internal consistency (i.e., scale reliability) for all 15 COVID-19 survey (α= 0.7; acceptable reliability). In light of the novel nature of our data collection instrument, we determined the scale to be acceptably reliable.[16] The COVID-19 section contained three measurement domains: 1) knowledge & awareness, 2) perceived threat to health, and 3) prevention behaviors. COVID-19 items were developed based on available guidance from the WHO and CDC and were reviewed by French, English, and Wolof speaking team members. The survey was translated from English to French by study team members in Senegal (MF) and UCLA (RN).

Response options were binary (e.g., agree/disagree) or Likert-style (i.e., ordinal). COVID-19 response items were aggregated into distinct scales based on the three measurement domains. Each scale ranged from 0 to 15. As the COVID-19 items were novel, these "scales" were used as a proxy measure for determining the degree of knowledge and awareness, perceived risk, and practicing of known prevention behaviors including 1) handwashing, 2) using hand sanitizer, 3) wearing a mask, 4) social distancing, and 5) staying at home. Perceived COVID-19 risk items drew from the Health Belief Model including perceived susceptibility ("Are you afraid of catching COVID-19?"; "Do you think the COVID-19 epidemic poses a risk to Africans?"), perceived severity ("Do you think COVID-19 represents a danger to your health?"; "How stressed do you feel about hearing about COVID-19?"), and perceived behavioral control ("If you thought you had caught COVID-19, would you know who to contact?").

Data Analysis

Descriptive statistics were generated for demographic attributes and COVID-19 items. Chi-squared tests assessed differences in demographic attributes between the

For peer review only

online and telephonic collection methods. Significance was determined at the alpha=0.05 level. For multivariate analysis, we recoded level of education, marital status, location (e.g., urban/rural), religion, and occupation to reduce the number of categories, creating binary or ordinal variables, as shown in Table 1. Participant age was collected as a continuous measure and recoded as both quartile age groups and as binary (above versus below median age). Median values were calculated for each of the three COVID-19 scales and used as thresholds to create binary variables (e.g., 0=low knowledge;1=high knowledge).

We built five logistic regression models that predicted COVID-19 behaviors individually (i.e., hand washing, mask wearing, social distancing, staying home) and in aggregate, the results of which are presented as Forest plots (Figure 2). For individual behavior models, dependent variables were whether or not participants practiced prevention behaviors daily in the past week; for the aggregate model the dependent variable was the prevention behavior scale ranging from 0 (practice no behaviors in past week) to 15 (practice all behaviors daily). Independent variables in each model included demographic items, perceived threat (high/low), and knowledge & awareness (high/low). All statistical analyses were conducted using Stata IC software (version 15). Forest plots were generated using the 'forestplot' package in RStudio software.[17]

Patient and Public Involvement

This study is a cross-sectional analysis of surveys conducted in 2020 using a multi-modal recruitment strategy. No patients were involved in our study. All analyses were conducted in collaboration with research partners in Dakar, Senegal so it was not

For peer review only - http://bmjopen.bmj.com/site/about/guidelines.xhtml

appropriate to involve specific public communities or patients or in the design, conduct, reporting or dissemination plans.

Results

A total of 1,452 Senegalese adults participated in our cross-sectional survey about COVID-19. Table 1 presents demographics characteristics for the study sample. Table 2 presents univariate descriptive statistics for COVID-19 survey items. Table 3 presents unadjusted regression models and Table 4 presents adjusted regression models. Figure 2 presents a Forest plot of our logistic regression model predicting prevention behaviors.

Sample Characteristics

Most participants identified as Muslim (94.5%) and primarily lived in in an urban area (77.1%). Nearly half completed secondary education (high school or more; 49.9%). Most participants were single (55.4%). Participants' ages ranges from 18-44 years, with a median age of 26 years and mean of 28.0 years (SD=7.6; mean and median data not presented). The sample was evenly split between females and males (50.1% versus 49.9%). A third of participants worked in the informal sector (e.g., craftsperson, restauranteur; 34.9%); another third were currently students (31.8%). Most participants completed the survey via phone rather than online, respectively 84.1% and 15.9%.

We identified some differences between our two recruitment methods – i.e., online versus telephone (data not presented). Compared to participants recruited online, participants recruited telephonically were more likely to be older on average (telephone=28.5 years versus online=25.0; p<.001), live in rural versus urban settings

For peer review only - http://bmjopen.bmj.com/site/about/guidelines.xhtml

(23.0% versus 4.4%; p<.001), and have less than college education (16.1% versus 79.7%; p<.001). Half of telephone participants were male, compared to less than four in ten online participants (49.9% versus 39.4%; p=.003). Significant differences were also identified between recruitment methods for relationship status (p<.001), profession (p<.001), and religion (p<.001). Compared to telephone participants, online participants also had significantly higher COVID-19 knowledge and awareness (p<.001) and were significantly more likely to engage in prevention behaviors (p<.001).

Knowledge and Awareness

As shown in Table 2, of the 1,452 participants in our study sample less than 1% had not heard about COVID-19 (0.4%). More than nine in ten participants knew that hand washing (94.8%) and mask wearing in public (97.8%) were effective methods to prevent COVID-19 transmission and/or infection. About half were aware that staying at home (48.6%) or avoiding groups of more than ten people (50.8%) were COVID-19 prevention recommendations. Nearly four in five participants (79.4%) said that they would know who to contact (e.g., testing, healthcare) if they suspected themselves to have had COVID-19. The median knowledge score was 12.5 out of 15 (range=0 [lowest] to 15 [highest]; mean=11.8 / SD=2.6; data not presented).

Perceived Threat

As shown in Table 2, nine in ten participants (94.6%) perceived COVID-19 to be a very or somewhat serious danger to their health. Almost three-quarters (73.3%) said that COVID-19 posed a serious risk to Africans. Median perceived threat score was 11.6 out of 15 (mean=10.9 /SD=3.5; data not presented).

For peer review only - http://bmjopen.bmj.com/site/about/guidelines.xhtml

Prevention Behaviors

As shown in Table 2, nine in ten participants reported washing their hands multiple times per day (94.4%). Nearly eight in ten used hand sanitizer multiple times per day (77.4%). Approximately seven in ten participants wore masks every time they left their home (65.6%). Six in ten reported practicing social distancing all or most of the time (59.3%). Half of participants reported staying home all or most of the time (52.3%). Less than one in ten people went out as usual (6.2%) or ignored social distancing (5.7%). Less than one in one hundred participants never wore a mask in public (0.4%). The median prevention behaviors score was 11 out of 15 (mean=10.5/ SD=2.3; data not presented). Unadjusted bivariate regression models are presented in Table 3 for reference. Below we discuss results for adjusted regression models shown in Table 4 and Figure 2;

Regression Analysis COVID-19 behaviors were significantly associated with COVID-19 beliefs and participant demographic attributes (see Figure 2, Table 4). We also observed differences in behaviors based on our participant recruitment method. Compared to participants recruited telephonically, participants recruited online were significantly more likely to socially distance (OR=3.33; p=<.001), wear masks (OR=3.26; p=.007), and stay home (OR=1.63; p=.013).

Participants who reported high degrees of perceived threat about COVID-19 were significantly more likely to practice prevention behaviors in both the aggregate and individual models (OR=1.69; p<.001). Knowledge and awareness related to COVID-19 poorly predicted practicing prevention behaviors, although high levels of knowledge and awareness were positively associated with participants' use of hand sanitizer (OR=1.47;

p=.035). At the same time, higher levels of knowledge and awareness about COVID-19 was negatively associated with staying home (OR=0.64; *p*=.002).

Compared to participants with less than a high school degree, participants with high school education or above were more likely to stay home (OR=1.67; p<.001), practice social distancing (OR=1.28; p=.070), and use hand sanitizer (OR=1.35; p=.062). Compared to urban participants, rural participants were more likely to socially distance (OR=1.36; p=.032), stay home (OR=1.80; p<.001), and wear a mask (OR=2.17; p=.021), but less likely to user hand sanitizer (OR=0.67; p=.010). Use of hand sanitizer was more likely among formal workers compared to students (OR=2.18; p=.002). Compared to participants less than 26 years old, older participants (over 26) were significantly less likely to stay home (OR=0.76; *p*=.045).

Sex-based differences were observed for COVID-19 prevention behaviors. Men had 41% lower odds of practicing *all five* COVID-19 prevention behaviors compared to women (OR=0.59; p<.001). Men also reported lower adherence to COVID-19 prevention measures for each of the five behaviors individually. Compared to women, men were 61% less likely to wash their hands on a daily basis (p<.001), 51% less likely to report staying home (p<.001), 39% less likely to wear masks daily (p<.037), 38% less likely to practice social distancing (p<.001), and 37% less likely to use hand sanitizer (p=.001).

For peer review only

Table 1. Sample characteristics. N=1,452. Data collected via phone and online surveys from Senegalese adults between June-August 2020.

Characteristic	Category	#	%
Survey modality	Telephone	1,221	84.1
	Online	231	15.9
Age Quartile	<21	353	24.3
	22-25	352	24.2
	26-34	354	24.4
	35+	393	27.1
Sex	Female	752	51.8
	Male	700	48.2
Location	Urban	1,118	77.1
	Rural	291	20.1
	Urban + Rural	42	2.9
Education	Superior	381	26.2
	Secondary	344	23.7
	Primary	284	19.6
	Middle	231	15.9
	No	145	10.0
	Other	67	4.6
Marital status	Single	804	55.4
	Married - monogamous	460	31.7
	Married - polygamist	161	11.1
	Divorced	20	1.4
	Widower	7	0.5
Occupation	Informal sector	569	39.2
	Student	400	27.6
	Formal sector	225	15.5
	Other	112	7.7
	Housewife	96	6.6
	Retired, out of work	49	3.4
	Religious leader	1	0.1
Religion	Muslim	1,372	94.5
	Catholic	65	4.5
	Protestant / Evangelical	8	0.6
	No	3	0.2
	Other	3	0.2
	Traditionalist	1	0.1

For peer review only

Table 2. Frequency (#) and percent (%) for responses to COVID-19 questionnaire items. N=1,452 Senegalese adult participants. All responses collected between June-August 2020.

	#	%
**Knowledge and Awareness Items**		
Have you ever heard of COVID-19, also called Coronavirus?		
Yes	1,446	99.6
No	6	0.4
What protective measure against COVID-19 / coronavirus do you know?		
Stay at home	705	48.6
Wash your hands regularly	1,377	94.8
Wear a mask in public	1,420	97.8
Avoid meeting with groups of more than 10 people	738	50.8
**Prevention Behavior Items (Past Week)**		
Staying at home		
Partly (I try to limit time away from home but go out several times a week)	602	41.5
Most of the time (I go out about once a week)	476	32.8
All the time (I didn't leave the house)	282	19.5
I went out as much as usual	90	6.2
Wash your hands with soap and water		
Several times a day, even if they do not seem dirty	830	57.4
Every day, as soon as they are dirty after the toilet, the kitchen or the work	535	37.0
Very rarely / Never	44	3.0
Once a day approximately	38	2.6
Wear a mask when you leave your home		
Each time	951	65.6
Most of the time	403	27.8
Sometimes	90	6.2
Never / I don't have a mask	6	0.4
Use hand sanitizer gel		
Every day, but only in the places where it is needed	595	41.0
Several times a day	528	36.4
Rarely / never	215	14.8
About once a day	112	7.7
Practice social distancing (limit the number of other people)		
Most of the time (I go out rarely and keep a distance)	557	38.4
Partly (I don't limit my social interactions but I keep my distance)	507	35.0
All the time (I am confined to my home)	303	20.9
Never (I act as usual: I pass other people and I shake their hands)	83	5.7
**Perceived Threat**		
Do you think COVID-19 represents a danger to your health?		
Yes, very serious	1,152	79.6
Somewhat serious	217	15.0

For peer review only

No	78	5.4
Are you afraid of catching COVID-19?		
Very afraid	913	63.1
Relatively afraid	347	24.0
Not afraid	186	12.9
Do you think the COVID-19 epidemic poses a risk to Africans?		
Yes, a serious risk	1,061	73.3
Yes, but a reasonable risk	283	19.5
No, a low risk	104	7.2
If you thought you had caught COVID-19, would you know who to contact?		
Yes	1,149	79.4
No	298	20.6
How stressed do you feel about hearing about COVID-19?		
Somewhat stressed	586	40.5
Stressed a little	366	25.3
Stressed a lot	262	18.1
Not at all stressed	234	16.2

For peer review only

Table 3. Univariate logistic regression models predicting COVID-19 prevention behaviors (aggregate + individually). N=1,452. All estimates presented are not adjusted for other model variables. All responses collected between June-August 2020. Alpha = 0.05. OR = odds ratio.

	**All Behaviors**				**Using Hand Sanitizer**				**Social Distancing**			
Predictor	*OR*	*95% CI*		*p*	*OR*	*95% CI*		*p*	*OR*	*95% CI*		*p*
Online participant[1]	2.39	1.80	3.18	<.001	2.46	1.61	3.75	<.001	3.33	2.36	4.70	<.001
COVID Perceived threat	1.71	1.37	2.13	<.001	1.75	1.35	2.28	<.001	2.01	1.62	2.51	<.001
Rural[2]	1.25	0.96	1.63	0.104	0.56	0.42	0.75	<.001	1.14	0.87	1.48	0.334
High school or above[3]	0.76	0.61	0.95	<.001	0.78	0.59	1.01	<.01	0.70	0.56	0.87	<.001
Formal worker[4]	0.98	0.73	1.33	0.018	1.49	1.00	2.22	0.059	1.10	0.81	1.50	0.002
Student[4]	1.56	1.26	1.94	0.901	1.54	1.20	1.98	0.053	1.67	1.35	2.06	0.532
COVID KAP	1.47	1.16	1.86	<.01	2.13	1.56	2.90	<.001	1.55	1.22	1.97	<.001
Single[5]	0.92	0.74	1.14	0.446	1.07	0.83	1.37	0.607	0.96	0.78	1.19	0.722
26 years or older[6]	0.80	0.64	0.99	0.044	1.09	0.85	1.39	0.511	0.81	0.66	1.00	0.048
Male[6]	0.49	0.39	0.61	<.001	0.60	0.47	0.77	<.001	0.52	0.42	0.64	<.001

	**Staying Home**				**Hand Washing**				**Mask Wearing**			
Predictor	*OR*	*95% CI*		*p*	*OR*	*95% CI*		*p*	*OR*	*95% CI*		*p*
Online participant[1]	1.63	1.22	2.17	<.01	2.49	1.07	5.78	0.029	2.17	1.04	4.53	0.036
COVID Perceived threat	1.63	1.32	2.01	<.001	3.86	2.11	7.05	<.001	3.16	1.87	5.34	<.001
Rural[2]	1.65	1.27	2.15	<.001	1.35	0.74	2.48	0.329	2.01	1.06	3.82	0.030
High school or above[3]	0.83	0.67	1.03	<.001	1.06	0.66	1.68	0.640	1.40	0.94	2.07	0.517
Formal worker[4]	0.87	0.65	1.17	0.093	1.48	0.73	3.00	0.814	1.93	1.05	3.56	0.094
Student[4]	1.71	1.39	2.11	0.348	0.90	0.58	1.40	0.273	0.87	0.58	1.32	0.035
COVID KAP	0.91	0.73	1.15	0.439	2.41	1.29	4.49	0.005	1.55	0.93	2.56	0.090
Single[5]	1.00	0.81	1.23	0.985	0.59	0.37	0.95	0.028	0.54	0.35	0.85	0.006
26 years or older[6]	0.74	0.60	0.91	0.005	1.53	0.98	2.41	0.063	1.39	0.92	2.11	0.119
Male[6]	0.45	0.37	0.56	<.001	0.34	0.21	0.56	<.001	0.54	0.35	0.82	0.004

[1]Ref=Telephone participant; [2]Ref=Urban; [3]Ref=Below high school; [4]Ref=Informal worker, housewife, retired, out of work, religious leader, other; [5]Ref=Married (monogamous + polygamous), divorced, widower; [6]Ref=Female

For peer review only - http://bmjopen.bmj.com/site/about/guidelines.xhtml

Table 4. Multivariate logistic regression models predicting COVID-19 prevention behaviors (aggregate + individually). N=1,452. All estimates presented are adjusted for other model variables. All responses collected between June-August 2020. Alpha = 0.05. aOR = adjusted odds ratio.

	**All Behaviors**				**Using Hand Sanitizer**				**Social Distancing**			
Predictor	*aOR*	*95% CI*		*p*	*aOR*	*95% CI*		*p*	*aOR*	*95% CI*		*p*
Online participant[1]	1.93	1.29	2.87	0.001	1.62	0.94	2.78	0.079	3.20	2.05	5.00	<.001
COVID Perceived threat	1.69	1.33	2.13	<.001	1.69	1.28	2.22	<.001	2.03	1.61	2.57	<.001
Rural[2]	1.49	1.12	1.98	0.006	0.67	0.50	0.91	0.010	1.36	1.03	1.81	0.032
High school or above[3]	1.33	1.01	1.76	0.044	1.35	0.98	1.84	0.062	1.28	0.98	1.67	0.070
Formal worker[4]	1.28	0.86	1.90	0.230	2.18	1.32	3.60	0.002	1.42	0.95	2.13	0.090
Student[4]	1.07	0.76	1.52	0.691	1.33	0.90	1.97	0.149	0.98	0.70	1.38	0.927
COVID KAP	0.96	0.72	1.29	0.798	1.47	1.03	2.10	0.035	0.89	0.66	1.18	0.411
Single[5]	0.75	0.56	1.02	0.070	1.20	0.86	1.69	0.281	0.79	0.59	1.06	0.116
26 years or older[6]	0.75	0.56	1.00	0.054	1.19	0.86	1.64	0.287	0.78	0.59	1.03	0.077
Male[6]	0.59	0.46	0.75	<.001	0.64	0.49	0.84	0.001	0.62	0.49	0.79	<.001
	**Staying Home**				**Hand Washing**				**Mask Wearing**			
Predictor	*aOR*	*95% CI*		*p*	*aOR*	*95% CI*		*p*	*aOR*	*95% CI*		*p*
Online participant[1]	1.62	1.09	2.42	0.018	2.48	0.89	6.96	0.084	3.25	1.31	8.04	0.011
COVID Perceived threat	1.62	1.29	2.03	<.001	3.09	1.67	5.71	<.001	2.81	1.64	4.82	<.001
Rural[2]	1.80	1.35	2.38	<.001	1.52	0.81	2.86	0.189	2.17	1.12	4.19	0.021
High school or above[3]	1.67	1.28	2.17	<.001	0.85	0.49	1.45	0.546	0.82	0.50	1.36	0.448
Formal worker[4]	0.98	0.67	1.44	0.918	1.54	0.67	3.53	0.311	2.44	1.03	5.75	0.042
Student[4]	0.89	0.64	1.25	0.510	1.12	0.58	2.16	0.733	1.10	0.60	1.99	0.760
COVID KAP	0.64	0.48	0.85	0.002	1.78	0.88	3.63	0.110	1.14	0.64	2.04	0.654
Single[5]	0.85	0.64	1.13	0.260	0.93	0.50	1.71	0.809	0.73	0.41	1.29	0.277
26 years or older[6]	0.76	0.58	0.99	0.045	1.40	0.80	2.46	0.243	1.05	0.63	1.77	0.847
Male[6]	0.49	0.39	0.62	<.001	0.39	0.23	0.66	<.001	0.61	0.39	0.97	0.037

[1]Ref=Telephone participant; [2]Ref=Urban; [3]Ref=Below high school; [4]Ref=Informal worker, housewife, retired, out of work, religious leader, other; [5]Ref=Married (monogamous + polygamous), divorced, widower; [6]Ref=Female

For peer review only - http://bmjopen.bmj.com/site/about/guidelines.xhtml

Discussion

In our cross-sectional sample of Senegalese adults, we observed that the majority of participants practiced COVID-19 prevention behaviors on a daily basis. Mask wearing, hand washing, and use of hand sanitizer were most frequently reported. Social distancing and staying at home were also reported albeit to a lower degree. We also identified a range of psychosocial and demographic predictors for COVID-19 prevention behaviors. For example, men and women differed in the degree to which they practiced COVID-19 prevention behaviors. Participants who perceived COVID-19 to be a threat were significantly more likely to practice every prevention behavior measured than those who did not perceive COVID-19 to be a threat. This is in line with existing health behavior research regarding perceived threat as a necessary precursor to behavior change.[14,15] Our findings from Senegal not only contribute to a growing body of literature about COVID-19 beliefs and behaviors in developing nations, but also support prior research in both developing and developed nations about predictors of health behaviors.

Prevention Behaviors

Men were less likely than women to report practicing any of the prevention behaviors that we studied. In the US, a developed nation, previous research shows that women are more likely to engage generally in preventive health behaviors adhering to public health recommendations, such as cancer screenings, exercise, and dietary intake.[18,19] Specific to COVID-19, women have also been shown to more frequently practice prevention behaviors like mask wearing, and this is true both in developed nations like the US,[20,21] as well as in developing nations like South Africa, [22]

For peer review only

Chile,[23] Turkey,[24], and China.[25] However, studies in Sierra Leone and Cameroon have both found that women were less likely to engage in COVID-19 prevention behaviors, contradictory to our findings from Senegal. In general, men may also be more likely to engage in risk taking behaviors,[26] whereas women are more risk averse.[27] Future research into adherence of recommended COVID-19 prevention behaviors should explore gender-based differences in risk perceptions and tolerance.

Practicing COVID-19 prevention behaviors was also positively associated with living in a rural versus urban area. It is possible that people residing in rural areas have an easier time implementing preventive behaviors such as staying at home and social distancing than people in more densely populated urban areas. Finally, Participants with at least a high school education were also more likely to engage in preventive behaviors. Educational differences in adoption of COVID-19 preventive health behaviors has previously been reported in studies in other developing nations like South Africa and China. [25,28,29] Such demographic variations in practicing COVID-19 prevention behaviors indicate priority populations for future health communication campaigns. For example, people in urban areas may require more prompting and resources to implement preventive behaviors in the future.

Considering our multi-modal recruitment strategy, we found that respondents from the online sample demonstrated greater knowledge and awareness of COVID-19 and engaged in prevention behaviors more often compared to respondents in the telephone sample, suggesting that strategies leveraging online communications and communities of users will be important to continue supporting and promoting prevention behaviors, in particular to build vaccine confidence as vaccine availability increases

For peer review only

throughout the region. Differences between respondents' based on recruitment method could indicate similar differences in Senegalese adults in general depending on whether they had access to and were frequent users of the internet. This provides support for both digital and on-the-ground messaging and communication to strengthen pro-prevention beliefs, attitudes, and behaviors. For example, health promotion campaigns could engage local social media influencers or bloggers to promote positive attitudes towards COVID-19 prevention behaviors, including vaccination, and rely on their networks of users to further disseminate health communications via word of mouth to their families and friends online and offline.

Health Beliefs

Our study findings comport with a robust body of evidence about COVID-19 knowledge, awareness, attitudes, and practices. In Senegal, we observed that knowledge, perceived risk, and practicing prevention behaviors for COVID-19 were very high in general – albeit with some demographic variations. A 2020 international review by Puspitasari and colleagues reported generally high knowledge and awareness about COVID-19, pro-prevention attitudes, and high adherence to COVID-19 prevention behaviors like handwashing and social distancing.[30] Closer to the West African and Senegalese contexts, Aduh and colleagues (2020) reported that in sub-Saharan Africa moderate to high levels of COVID-19 knowledge and perceived risk.[12]

We did not find an association between knowledge and engaging in prevention behaviors, a finding both contrary to and in line with prior international research of infectious disease practices in developing nations. For example, a 2020 study in Guyana found that knowledge was a significant predictor of practicing malaria and

For peer review only - http://bmjopen.bmj.com/site/about/guidelines.xhtml

dengue prevention behaviors but not for Zika virus and leishmaniosis, a common protozoan-caused skin infection.[31] Specific to COVID-19, a 2020 study in China reported that participants with increased knowledge were more likely to go to crowded places and less likely to wear masks.[25] Finally, a 2021 scoping review of 28 studies on COVID-19 beliefs and preventive practices in 9 sub-Saharan African nations – not including Senegal – reported that although knowledge about COVID-19 was generally high, it was not always associated with increased risk perceptions or attitudes towards the virus, and by extension was a poor predictor of prevention behaviors.[32]

In the current study, we found that perceived COVID-19 risk was a stronger predictor of prevention behaviors, which is also supported by previous international research.[2,33] Low perceptions of risk may result in low compliance with virus restrictions and adherence to prevention behaviors,[34–36] and therefore facilitates COVID-19's spread. This confirms another recent study which recruited using social media from the United States, China, Taiwan, and Mexico.[2] Hsing and colleagues found that perceived COVID-19 severity and susceptibility – constructs that comprise perceived risk/threat in the Health Belief Model – were both predictors of hand washing and social distancing.[2] In our study based in Senegal, perceived risk not only predicted hand washing and social distancing, it also predicted staying home, using hand sanitizer, and mask wearing. These findings suggest that to prevent COVID-19 spread in Senegal and other parts of francophone West Africa, we need to focus more on perceived risk than knowledge. For example, health agencies and organizations should promote health communications that highlight Senegalese residents' susceptibility to contracting COVID-19 as well as its potential health impacts. Messaging

For peer review only - http://bmjopen.bmj.com/site/about/guidelines.xhtml

should be targeted towards groups that we identified as having lower adherence to prevention behaviors, in particular men.

A Focus on Senegal

In general, Senegalese participants had high levels of COVID-19 knowledge and awareness, perceived COVID-19 to be a threat both personally and across Senegal, and in most cases adopted known prevention behaviors on a daily or regular basis. Looking forward, as vaccines become more widely available in Senegal and many West African nations, inequities in access will make sustaining preventive behaviors paramount to continued control of the virus.[37] Our findings suggest that other nations should look to Senegalese communications about COVID-19 to learn messages that may resonate more effectively with their populations to increase prevention behaviors.

As has been stated, most participants reported moderate to high adherence to COVID-19 prevention behaviors. To explain our findings, we must consider lessons learned from previous regional epidemics and how they relate to evolving social contexts (e.g., norms, culture, population attributes), in particular Ebola.[38] Many behaviors associated with preventing COVID-19 were already familiar to residents of African nations who experienced Ebola outbreaks, including hand washing, use of personal protective equipment, and social distancing.[39] COVID-19 in West Africa comes several years after the Ebola outbreak in 2014-2015, which not only tested the limits of the health systems but also demonstrated its resilience. For example, although Senegal experiences a nationwide shortage of doctors – just 7 per 100,000 persons in 2020 – the nation has been comparatively effective at controlling COVID-19 infections. The first case of COVID-19 in Senegal, a country of 17 million, was reported in early

For peer review only - http://bmjopen.bmj.com/site/about/guidelines.xhtml

March 2020, prompting the Ministry of Health to develop protocols to monitor and address cases within Senegal and at its borders and airports. Cases in Senegal remained low throughout Spring, but like other nations rose considerably in the latter half of 2020 and first months of 2021.[40,41] On August 8th, 2020, Senegal's Minister of Interior mandated mask wearing in indoor spaces, banned gatherings in public spaces like beaches, theatres, and sporting grounds, and prohibited demonstrations on public highways.[42] These restrictions were emphasized in particular for the densely populated region of Dakar, but did not include mandatory lockdowns, local travel restrictions, or school/university closures. Although our survey was conducted in summer 2020, our findings continue to provide relevant context to current COVID-19 epidemiological data in Senegal: as of January 27, 2022, the 7-day rolling average for COVID-19 cases per million people was lower in Senegal than both the rest of Africa and the world – 7.95 versus 22.65 versus 427.1, respectively.[43]

Senegal's success at controlling the virus throughout 2020 has been attributed to many factors, including a citizenry who has witnessed the devastating impacts of the Ebola epidemic. More broadly across West Africa, citizens may be more likely to comply with public health restrictions because of their experience with Ebola,[44] and this extends to individual behavior changes as well. For example, one study reported that handwashing was shown to increase in Senegal as a result of Ebola education campaigns.[45] In our analysis, we found that daily hand washing was the prevention behavior most often reported by participants. Future health campaigns may look at Ebola prevention messages to identify behaviors and information that may be reinforced in the context of COVID-19.

For peer review only - http://bmjopen.bmj.com/site/about/guidelines.xhtml

Limitations

- Our findings may not be generalizable beyond Senegal, or more specifically Senegalese adults, because our targeted recruitment strategy.
- The recruitment strategy increased the potential for selection bias because participants were recruited online and on-the-ground in Senegal; thus, all participants, by the nature of the recruitment methods, had access to the internet and/or a cell phone.
- We did not identify significant relationships for many factors in our regression model because our cross-sectional study was not statistically powered to detect significant differences and we did not assess the discriminatory power of our novel questionnaire items or scales.
- Staying at home or social distancing prevention behaviors may have been confounded by other factors not included in our regression modelling, in particular government lockdowns, local travel restrictions, school/university closures, and whether a participant worked outside of the home or not.
- Although our COVID-19 survey items were developed by the study team based on available WHO guidance, they were not validated tools like the ICP COVID survey and therefore have limited transferability to similar investigations.

Conclusion

Our study contributes to a growing body of literature about COVID-19 in Africa,[46,47] and is the first study to look specifically at knowledge and prevention behaviors in the francophone West African nation of Senegal. In Senegal, we observed that knowledge and perceived risk of COVID-19 were very high in general. But, risk was

For peer review only

a stronger and more influential predictor of COVID-19 prevention behaviors. COVID-19 prevention behaviors also varied by sex, education, location, and country. These findings suggest that in West Africa, nations should seek to elevate risk perceptions about COVID-19 and develop messaging to better target certain groups. Elevating the perceived risk of a population is a difficult issue because public health programs do not want to raise unnecessary alarm. Thus, messages around risk of COVID-19 should be accompanied by empowering messages of how individuals can protect themselves, their families, and their communities. Health campaigns should look backwards to successes with Ebola for improving health messaging about COVID-19, and may also look to replicate the successes of regional examples like Senegal.

For peer review only

Figure 1. Study recruitment and sampling diagram.

Figure 2. Forest plot diagrams for logistic regression models predicting COVID-19 prevention behaviors (aggregate + individually). Square box represents point estimate and horizontal bar represents 95% CI. Confidence intervals that cross OR threshold of 1.0 are not statistically significant at the alpha=0.05 level. All estimates presented are adjusted for other model variables. N=1,452. All responses collected between June-August 2020.

Data availability statement

No data are available.

Ethics statements

Patient consent for publication: Not required.

Research ethics approval: All study procedures and protocols were approved by Drexel University's institutional ethical review board (Protocol ID: 00015164). Written informed consent was obtained from all participants.

Acknowledgements

We are grateful for support from our French and Senegalese collaborators in Dakar, Senegal at The African Health and Education Network (NGO RAES) and at Université Cheikh-Anta-Diop. Data collection was completed while the first (MK) and last author (PM) were affiliated with the Drexel University Dornsife School of Public Health, and while the second author (MB) was a graduate student at UCLA Fielding School of Public Health. Finally, we are thankful to all participants in this study.

Competing Interests

None declared.

Funding

This study received funding from the Bill & Melinda Gates Foundation (OPP1181104). Disclaimer. The study sponsors had no role in study design, data collection, data analysis, our decision to publish, or preparing the manuscript.

Contributors

All authors contributed to the editing and reviewing of this paper. MK, MB, DG, and PM were involved in conceptualizing this article. MF and RN led development of the data collection tool. MK and RN designed analyses for this manuscript, and MK conducted the analyses. MK, MB, DG, and PM wrote the paper. All authors reviewed, edited, and revised the paper.

For peer review only

References

1 Naidoo AV, Hodkinson P, Lai King L, *et al.* African authorship on African papers during the COVID-19 pandemic. *BMJ Glob Health* 2021;**6**:e004612. doi:10.1136/bmjgh-2020-004612

2 Hsing JC, Ma J, Barrero-Castillero A, *et al.* Influence of Health Beliefs on Adherence to COVID-19 Preventative Practices: International, Social Media–Based Survey Study. *J Med Internet Res* 2021;**23**:e23720. doi:10.2196/23720

3 Alistair Mallard, Maria Amalia Pesantes, Carol Zavaleta-Cortijo, *et al.* An urgent call to collect data related to COVID-19 and Indigenous populations globally. *BMJ Glob Health* 2021;**6**:e004655. doi:10.1136/bmjgh-2020-004655

4 Sarah Wehbe, Fahme SA, Rizk A, *et al.* COVID-19 in the Middle East and North Africa region: an urgent call for reliable, disaggregated and openly shared data. *BMJ Glob Health* 2021;**6**:e005175. doi:10.1136/bmjgh-2021-005175

5 Bodewes AJ, Kunst AE. Involving hard-to-reach ethnic minorities in low-budget health research: lessons from a health survey among Moluccans in the Netherlands. *BMC Res Notes* 2016;**9**:319. doi:10.1186/s13104-016-2124-1

6 Dusek G, Yurova Y, P. Ruppel C. Using Social Media and Targeted Snowball Sampling to Survey a Hard-to-reach Population: A Case Study. *Int J Dr Stud* 2015;**10**:279–99. doi:10.28945/2296

7 Wilkerson JM, Iantaffi A, Grey JA, *et al.* Recommendations for Internet-Based Qualitative Health Research With Hard-to-Reach Populations. *Qual Health Res* 2014;**24**:561–74. doi:10.1177/1049732314524635

8 BBC News. Coronavirus in Senegal: Keeping Covid-19 at bay. BBC News. 2020.https://www.bbc.com/news/world-africa-54388340 (accessed 30 Mar 2021).

9 FP Analytics. The COVID-19 Global Response Index: A country-by-country assessment of government responses to the pandemic. Foreign Policy. 2021.https://globalresponseindex.foreignpolicy.com/ (accessed 30 Mar 2021).

10 Chitungo I, Dzobo M, Hlongwa M, *et al.* COVID-19: Unpacking the low number of cases in Africa. *Public Health Pract* 2020;**1**:100038. doi:10.1016/j.puhip.2020.100038

11 Mbow M, Lell B, Jochems SP, *et al.* COVID-19 in Africa: Dampening the storm? *Science* 2020;**369**:624–6. doi:10.1126/science.abd3902

12 Aduh U, Folayan MO, Afe A, *et al.* Risk perception, public health interventions, and Covid-19 pandemic control in sub-saharan Africa. *J Public Health Afr* Published Online First: 9 October 2020. doi:10.4081/jphia.2021.1622

For peer review only - http://bmjopen.bmj.com/site/about/guidelines.xhtml

13 Worldometer. Realtime Tracker: Population of Western Africa. 2021.https://www.worldometers.info/world-population/western-africa-population/ (accessed 22 Apr 2021).

14 Rosenstock IM. Historical Origins of the Health Belief Model. *Health Educ Monogr* 1974;**2**:328–35. doi:10.1177/109019817400200403

15 Glanz K, Rimer BK, Viswanath K. *Health Behavior: Theory, Research, and Practice*. John Wiley & Sons 2015.

16 DeVillis RF. Scale development: Theory and applications. 1991.

17 Gordon M, Lumley T, Gordon MM. Package 'forestplot'. *Adv For Plot Using 'grid'Graphics* 2016.

18 Berrigan D, Dodd K, Troiano RP, *et al.* Patterns of health behavior in U.S. adults. *Prev Med* 2003;**36**:615–23. doi:10.1016/S0091-7435(02)00067-1

19 Liang W, Shediac-Rizkallah MC, Celentano DD, *et al.* A population-based study of age and gender differences in patterns of health-related behaviors. *Am J Prev Med* 1999;**17**:8–17. doi:10.1016/S0749-3797(99)00040-9

20 Naeim A, Baxter-King R, Wenger N, *et al.* Effects of Age, Gender, Health Status, and Political Party on COVID-19–Related Concerns and Prevention Behaviors: Results of a Large, Longitudinal Cross-sectional Survey. *JMIR Public Health Surveill* 2021;**7**:e24277. doi:10.2196/24277

21 Okten IO, Gollwitzer A, Oettingen G. Gender differences in preventing the spread of coronavirus. *Behav Sci Policy* 2020;**6**:14.

22 Majam M, Fischer A, Phiri J, *et al.* International citizen project to assess early stage adherence to public health measures for COVID-19 in South Africa. *PLOS ONE* 2021;**16**:e0248055. doi:10.1371/journal.pone.0248055

23 Bronfman N, Repetto P, Cordón P, *et al.* Gender Differences on Psychosocial Factors Affecting COVID-19 Preventive Behaviors. *Sustainability* 2021;**13**:6148. doi:10.3390/su13116148

24 Yıldırım M, Geçer E, Akgül Ö. The impacts of vulnerability, perceived risk, and fear on preventive behaviours against COVID-19. *Psychol Health Med* 2021;**26**:35–43. doi:10.1080/13548506.2020.1776891

25 Zhong B-L, Luo W, Li H-M, *et al.* Knowledge, attitudes, and practices towards COVID-19 among Chinese residents during the rapid rise period of the COVID-19 outbreak: a quick online cross-sectional survey. *Int J Biol Sci* 2020;**16**:1745–52. doi:10.7150/ijbs.45221

For peer review only

26 Byrnes JP, Miller DC, Schafer WD. Gender differences in risk taking: A meta-analysis. *Psychol Bull* 1999;**125**:367–83. doi:10.1037/0033-2909.125.3.367

27 Adhikari B, O'Leary VE. Gender Differences in Risk Aversion: A Developing Nation's Case. Rochester, NY: : Social Science Research Network 2011. https://papers.ssrn.com/abstract=1522974 (accessed 9 Sep 2021).

28 Chen X, Chen H. Differences in Preventive Behaviors of COVID-19 between Urban and Rural Residents: Lessons Learned from A Cross-Sectional Study in China. *Int J Environ Res Public Health* 2020;**17**:4437. doi:10.3390/ijerph17124437

29 Kollamparambil U, Oyenubi A. Behavioural response to the Covid-19 pandemic in South Africa. *PLOS ONE* 2021;**16**:e0250269. doi:10.1371/journal.pone.0250269

30 Puspitasari IM, Yusuf L, Sinuraya RK, *et al.* Knowledge, Attitude, and Practice During the COVID-19 Pandemic: A Review. J. Multidiscip. Healthc. 2020;**13**:727–33. doi:10.2147/JMDH.S265527

31 Aerts C, Revilla M, Duval L, *et al.* Understanding the role of disease knowledge and risk perception in shaping preventive behavior for selected vector-borne diseases in Guyana. *PLoS Negl Trop Dis* 2020;**14**:e0008149. doi:10.1371/journal.pntd.0008149

32 Nwagbara UI, Osual EC, Chireshe R, *et al.* Knowledge, attitude, perception, and preventative practices towards COVID-19 in sub-Saharan Africa: A scoping review. *PLOS ONE* 2021;**16**:e0249853. doi:10.1371/journal.pone.0249853

33 Li S, Feng B, Liao W, *et al.* Internet Use, Risk Awareness, and Demographic Characteristics Associated With Engagement in Preventive Behaviors and Testing: Cross-Sectional Survey on COVID-19 in the United States. *J Med Internet Res* 2020;**22**:e19782. doi:10.2196/19782

34 Chertok IRA. Perceived risk of infection and smoking behavior change during COVID-19 in Ohio. *Public Health Nurs* 2020;**n/a**. doi:10.1111/phn.12814

35 Gierlach E, Belsher BE, Beutler LE. Cross-cultural differences in risk perceptions of disasters. *Risk Anal Off Publ Soc Risk Anal* 2010;**30**:1539–49. doi:10.1111/j.1539-6924.2010.01451.x

36 Slovic P, Fischhoff B, Lichtenstein S. Why Study Risk Perception? *Risk Anal* 1982;**2**:83–93. doi:10.1111/j.1539-6924.1982.tb01369.x

37 Reuters. 'Immense joy' as Senegal begins public vaccine distribution. Reuters. 2021.https://www.reuters.com/article/us-health-coronavirus-senegal-vaccinatio-idUSKBN2AO2OH (accessed 30 Mar 2021).

38 Bavel JJV, Baicker K, Boggio PS, *et al.* Using social and behavioural science to support COVID-19 pandemic response. *Nat Hum Behav* 2020;**4**:460–71. doi:10.1038/s41562-020-0884-z

For peer review only

39 Gholizadeh P, Sanogo M, Oumarou A, *et al.* Fighting COVID-19 in the West Africa after experiencing the Ebola epidemic. *Health Promot Perspect* 2021;**11**:5–11. doi:10.34172/hpp.2021.02

40 Dia N, Lakh NA, Diagne MM, *et al.* COVID-19 Outbreak, Senegal, 2020. *Emerg Infect Dis* 2020;**26**:2771–3. doi:10.3201/eid2611.202615

41 Dong E, Du H, Gardner L. An interactive web-based dashboard to track COVID-19 in real time. *Lancet Infect Dis* 2020;**20**:533–4. doi:10.1016/S1473-3099(20)30120-1

42 Crisis24. Senegal: Authorities reintroduce COVID-19 measures following spike in cases. 2020.https://crisis24.garda.com/alerts/2020/08/senegal-authorities-reintroduce-covid-19-measures-following-spike-in-cases-august-8-update-12 (accessed 28 Jan 2022).

43 Our World in Data. COVID-19 Data Explorer. Our World Data. 2022.https://ourworldindata.org/coronavirus-data-explorer (accessed 28 Jan 2022).

44 Rosenthal PJ, Breman JG, Djimde AA, *et al.* COVID-19: Shining the Light on Africa. *Am J Trop Med Hyg* Published Online First: 5 May 2020. doi:10.4269/ajtmh.20-0380

45 Sougou NM, Diouf J, Leye MMM, *et al.* What community-based public health approaches in West Africa for COVID-19 epidemic? A reflection based on the African socio-cultural context. *Pan Afr Med J* 2020;**35**. doi:10.11604/pamj.supp.2020.35.2.24415

46 Gesesew HA, Koye DN, Fetene DM, *et al.* Risk factors for COVID-19 infection, disease severity and related deaths in Africa: a systematic review. *BMJ Open* 2021;**11**:e044618. doi:10.1136/bmjopen-2020-044618

47 Kana MA, LaPorte R, Jaye A. Africa's contribution to the science of the COVID-19/SARS-CoV-2 pandemic. *BMJ Glob Health* 2021;**6**:e004059. doi:10.1136/bmjgh-2020-004059

For peer review only

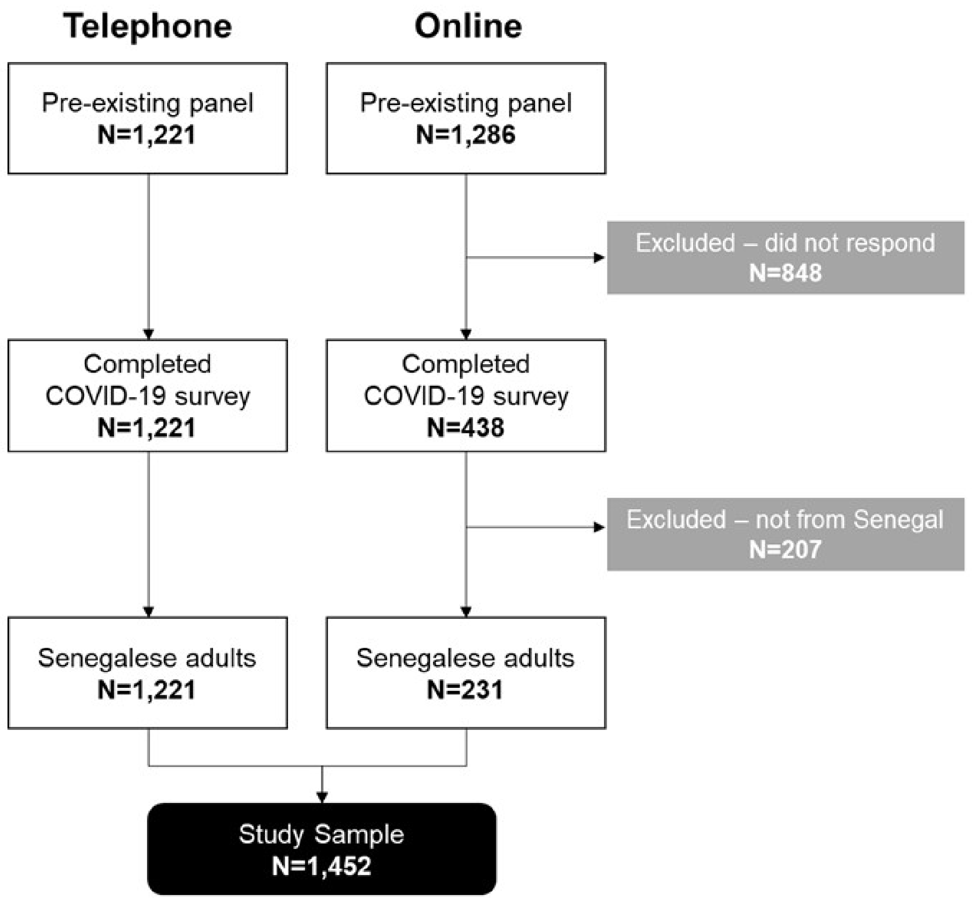

Figure 1. Study recruitment and sampling diagram.

184x166mm (96 x 96 DPI)

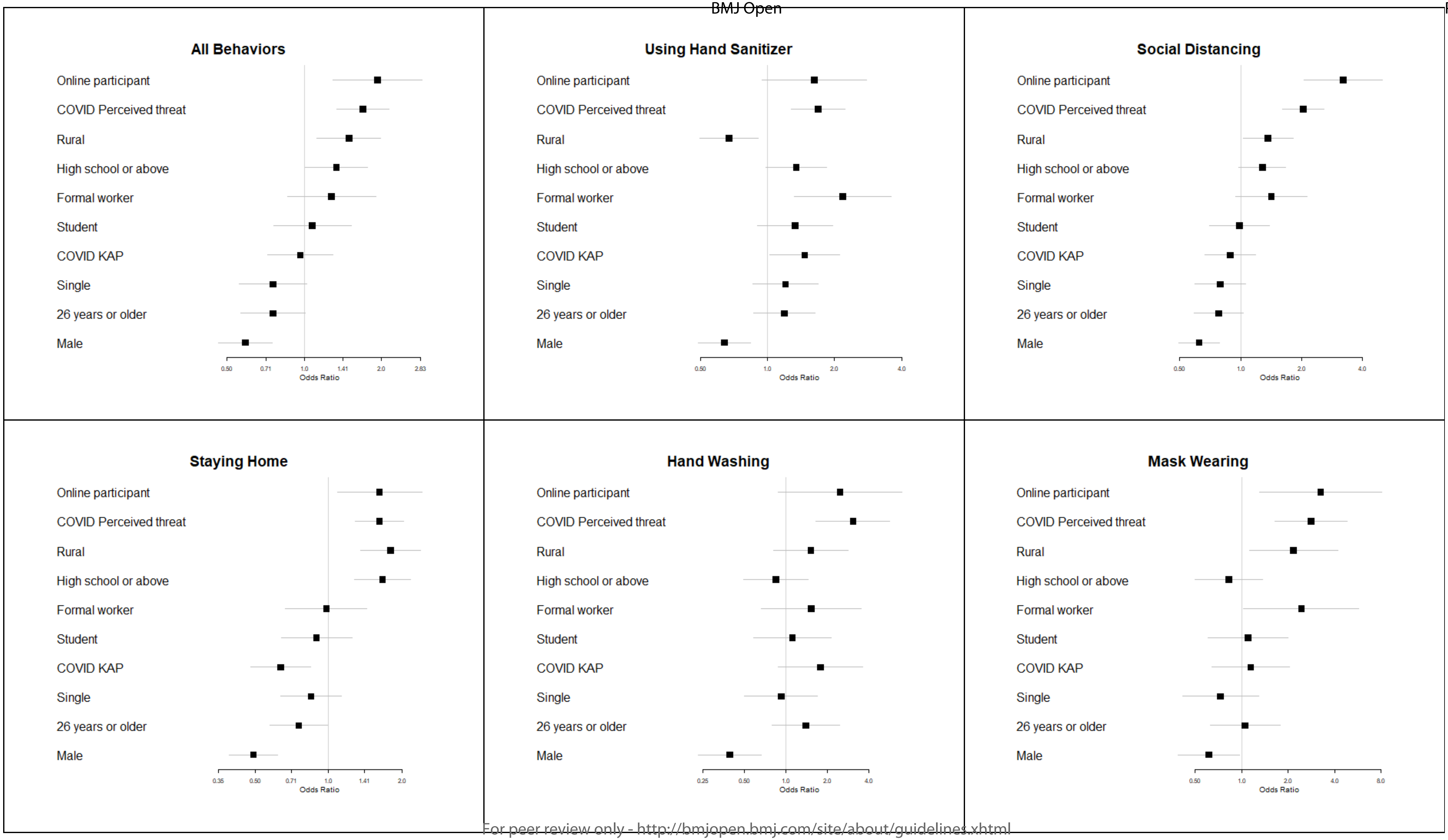
All Behaviors
Online participant
COVID Perceived threat
Rural
High school or above
Formal worker
Student
COVID KAP
Single
26 years or older
Male
0.50 0.71 1.0 1.41 2.0 2.83
Odds Ratio
Using Hand Sanitizer
Online participant
COVID Perceived threat
Rural
High school or above
Formal worker
Student
COVID KAP
Single
26 years or older
Male
0.50 1.0 2.0 4.0
Odds Ratio
Social Distancing
Online participant
COVID Perceived threat
Rural
High school or above
Formal worker
Student
COVID KAP
Single
26 years or older
Male
0.50 1.0 2.0 4.0
Odds Ratio
Staying Home
Online participant
COVID Perceived threat
Rural
High school or above
Formal worker
Student
COVID KAP
Single
26 years or older
Male
0.35 0.50 0.71 1.0 1.41 2.0
Odds Ratio
Hand Washing
Online participant
COVID Perceived threat
Rural
High school or above
Formal worker
Student
COVID KAP
Single
26 years or older
Male
0.25 0.50 1.0 2.0 4.0
Odds Ratio
Mask Wearing
Online participant
COVID Perceived threat
Rural
High school or above
Formal worker
Student
COVID KAP
Single
26 years or older
Male
0.50 1.0 2.0 4.0 8.0
Odds Ratio

Reporting checklist for cross sectional study.

Based on the STROBE cross sectional guidelines.

Instructions to authors

Complete this checklist by entering the page numbers from your manuscript where readers will find each of the items listed below.

Your article may not currently address all the items on the checklist. Please modify your text to include the missing information. If you are certain that an item does not apply, please write "n/a" and provide a short explanation.

Upload your completed checklist as an extra file when you submit to a journal.

In your methods section, say that you used the STROBE cross sectionalreporting guidelines, and cite them as:

von Elm E, Altman DG, Egger M, Pocock SJ, Gotzsche PC, Vandenbroucke JP. The Strengthening the Reporting of Observational Studies in Epidemiology (STROBE) Statement: guidelines for reporting observational studies.

For peer review only

		Reporting Item	Page Number
**Title and abstract**			
Title	#1a	Indicate the study’s design with a commonly used term in the title or the abstract	1

Abstract	#1b	Provide in the abstract an informative and balanced summary of what was done and what was found	2
**Introduction**			
Background / rationale	#2	Explain the scientific background and rationale for the investigation being reported	5-7
Objectives	#3	State specific objectives, including any prespecified hypotheses	7
**Methods**			
Study design	#4	Present key elements of study design early in the paper	8
Setting	#5	Describe the setting, locations, and relevant dates, including periods of recruitment, exposure, follow-up, and data collection	8
Eligibility criteria	#6a	Give the eligibility criteria, and the sources and methods of selection of participants.	8-9
	#7	Clearly define all outcomes, exposures, predictors, potential confounders, and effect modifiers. Give diagnostic criteria, if applicable	10-11
Data sources / measurement	#8	For each variable of interest give sources of data and details of methods of assessment (measurement). Describe comparability of	9-10

For peer review only

		assessment methods if there is more than one group. Give information separately for for exposed and unexposed groups if applicable.	
Bias	#9	Describe any efforts to address potential sources of bias	10-11
Study size	#10	Explain how the study size was arrived at	8-9
Quantitative variables	#11	Explain how quantitative variables were handled in the analyses. If applicable, describe which groupings were chosen, and why	10
Statistical methods	#12a	Describe all statistical methods, including those used to control for confounding	10-11
Statistical methods	#12b	Describe any methods used to examine subgroups and interactions	10-11
Statistical methods	#12c	Explain how missing data were addressed	9
Statistical methods	#12d	If applicable, describe analytical methods taking account of sampling strategy	11
Statistical methods	#12e	Describe any sensitivity analyses	N/A - Compared by recruitment method but didn't look at one specific group.

Results

For peer review only

Participants	#13a	Report numbers of individuals at each stage of study—eg numbers potentially eligible, examined for eligibility, confirmed eligible, included in the study, completing follow-up, and analysed. Give information separately for for exposed and unexposed groups if applicable.	12
Participants	#13b	Give reasons for non-participation at each stage	N/A - Only completed responses were included.
Participants	#13c	Consider use of a flow diagram	9
Descriptive data	#14a	Give characteristics of study participants (eg demographic, clinical, social) and information on exposures and potential confounders. Give information separately for exposed and unexposed groups if applicable.	12-13
Descriptive data	#14b	Indicate number of participants with missing data for each variable of interest	N/A - Only complete responses were included in the study sample.
Outcome data	#15	Report numbers of outcome events or summary measures. Give information separately for exposed and unexposed groups if applicable.	12-14

For peer review only

Main results	#16a	Give unadjusted estimates and, if applicable, confounder-adjusted estimates and their precision (eg, 95% confidence interval). Make clear which confounders were adjusted for and why they were included	14
Main results	#16b	Report category boundaries when continuous variables were categorized	11
Main results	#16c	If relevant, consider translating estimates of relative risk into absolute risk for a meaningful time period	N/A - Results estimates were reported as unadjusted and adjusted odds ratios.
Other analyses	#17	Report other analyses done—e.g., analyses of subgroups and interactions, and sensitivity analyses	12
**Discussion**			
Key results	#18	Summarise key results with reference to study objectives	21
Limitations	#19	Discuss limitations of the study, taking into account sources of potential bias or imprecision. Discuss both direction and magnitude of any potential bias.	27

For peer review only

Interpretation	#20	Give a cautious overall interpretation considering objectives, limitations, multiplicity of analyses, results from similar studies, and other relevant evidence.	27-28
Generalisability	#21	Discuss the generalisability (external validity) of the study results	27
**Other Information**			
Funding	#22	Give the source of funding and the role of the funders for the present study and, if applicable, for the original study on which the present article is based	29

Notes:

- 12e: N/A - Compared by recruitment method but didn't look at one specific group.
- 13b: N/A - Only completed responses were included.
- 14b: N/A - Only complete responses were included in the study sample.
- 16c: N/A - Results estimates were reported as unadjusted and adjusted odds ratios. The STROBE checklist is distributed under the terms of the Creative Commons Attribution License CC-BY. This checklist was completed on January 31, 2022.

For peer review only

BMJ Open

A cross-sectional study of COVID-19 knowledge, beliefs and prevention behaviors among adults in Senegal

Journal:	*BMJ Open*
Manuscript ID	bmjopen-2021-057914.R2
Article Type:	Original research
Date Submitted by the Author:	05-Apr-2022
Complete List of Authors:	Kearney, Matthew; University of Pennsylvania Perelman School of Medicine, Family Medicine and Community Health<br>Bornstein, Marta; The Ohio State University<br>Fall, Marieme; The African Health and Education Network (NGO RAES)<br>Nianogo, Roch; UCLA School of Public Health, Epidemiology<br>Glik, D; UCLA, Community Health Sciences<br>Massey, Philip; University of Arkansas Fayetteville, Health, Human Performance, and Recreation
**Primary Subject Heading**:	Public health
Secondary Subject Heading:	Global health, Infectious diseases, Communication
Keywords:	COVID-19, Public health < INFECTIOUS DISEASES, PUBLIC HEALTH

SCHOLARONE™
Manuscripts

BMJ

I, the Submitting Author has the right to grant and does grant on behalf of all authors of the Work (as defined in the below author licence), an exclusive licence and/or a non-exclusive licence for contributions from authors who are: i) UK Crown employees; ii) where BMJ has agreed a CC-BY licence shall apply, and/or iii) in accordance with the terms applicable for US Federal Government officers or employees acting as part of their official duties; on a worldwide, perpetual, irrevocable, royalty-free basis to BMJ Publishing Group Ltd ("BMJ") its licensees and where the relevant Journal is co-owned by BMJ to the co-owners of the Journal, to publish the Work in this journal and any other BMJ products and to exploit all rights, as set out in our licence.

The Submitting Author accepts and understands that any supply made under these terms is made by BMJ to the Submitting Author unless you are acting as an employee on behalf of your employer or a postgraduate student of an affiliated institution which is paying any applicable article publishing charge ("APC") for Open Access articles. Where the Submitting Author wishes to make the Work available on an Open Access basis (and intends to pay the relevant APC), the terms of reuse of such Open Access shall be governed by a Creative Commons licence – details of these licences and which Creative Commons licence will apply to this Work are set out in our licence referred to above.

Other than as permitted in any relevant BMJ Author's Self Archiving Policies, I confirm this Work has not been accepted for publication elsewhere, is not being considered for publication elsewhere and does not duplicate material already published. I confirm all authors consent to publication of this Work and authorise the granting of this licence.

For peer review only

Title

A cross-sectional study of COVID-19 knowledge, beliefs and prevention behaviors among adults in Senegal

Authors

Matthew D. Kearney PhD MPH – Department of Family Medicine and Community Health, Perelman School of Medicine, University of Pennsylvania, Philadelphia, Pennsylvania, USA, ORCID ID: 0000-0003-2898-573X

Marta Bornstein PhD MPH – College of Public Health, The Ohio State University, Columbus, OH, USA, ORCID ID: 0000-0002-5200-8181

Marieme Fall MPH – African Health and Education Network (RAES), Dakar, Senegal

Roch Nianogo PhD – Department of Epidemiology, UCLA Fielding School of Public Health, Los Angeles, CA, USA, ORCID ID: 0000-0001-5932-6169

Deborah Glik PhD – Department of Community Health Sciences, UCLA Fielding School of Public Health, Los Angeles, CA, USA, ORCID ID: 0000-0002-2532-3661

Philip M. Massey PhD MPH – Department of Health, Human Performance, and Recreation, College of Education and Health Professions, University of Arkansas, Fayetteville, AR, USA, ORCID: 0000-0002-0577-8618

Corresponding Author

Name: Dr. Matthew D. Kearney

Address: Penn Presbyterian Medical Center, Andrew Mutch Building, 51 N. 39th Street, Philadelphia, PA 19104

Email: kearnm@pennmedicine.upenn.edu

Word Count: 4,646

For peer review only

Abstract

Objectives: The aim of the study was to explore COVID-19 beliefs and prevention behaviors in a francophone West African nation, Senegal.

Design: This was a cross-sectional analysis of survey data collected via a multi-modal observational study.

Participants: Senegalese adults aged 18 years or older (n=1,452).

Primary and secondary outcome measures: Primary outcome measures were COVID-19 prevention behaviors. Secondary outcome measures included COVID-19 knowledge and beliefs. Univariate, bivariate, and multivariate statistics were generated to describe the sample and explore potential correlations.

Setting: Participants from Senegal were recruited online and telephonically between June-August 2020.

Results: Mask wearing, hand washing, and use of hand sanitizer were most frequently reported. Social distancing and staying at home were also reported albeit to a lower degree. Knowledge and perceived risk of COVID-19 were very high in general, but risk was a stronger and more influential predictor of COVID-19 prevention behaviors. Men, compared to women, had lower odds (AOR=0.59, 95% CI: 0.46-0.75, p<.001) of reporting prevention behaviors. Rural residents (vs. urban; AOR=1.49, 95% CI: 1.12-1.98, p=.001) and participants with at least a high school education (vs. less than high school education; AOR=1.33, 95% CI: 1.01-1.76, p=.006) were more likely to report COVID-19 prevention behaviors.

For peer review only

Conclusions: In Senegal, we observed high compliance with recommended COVID-19 prevention behaviors among our sample of respondents, in particular for masking and personal hygiene practice. We also identified a range of psychosocial and demographic predictors for COVID-19 prevention behaviors such as knowledge and perceived risk. Stakeholders and decision makers in Senegal and across Africa can use place-based evidence like ours to address COVID-19 risk factors and intervene effectively with policies and programming. Use of both phone and online surveys enhances representation and study generalizability and should be considered in future research with hard-to-reach populations.

Article Summary

Strengths and limitations of this study

- The main strength of our study was the use of a multi-modal data collection strategy, online and via telephone, which bolstered the representativeness of our total sample within Senegal and is a key strength of our study.
- We adapted pre-existing research cohorts and developed novel data collection instruments to capture information about COVID-19, and this may serve as a model for the responsiveness of ongoing scholarship to future global events.
- To address potential confounding between recruitment methods, we controlled for recruitment method in our multivariate regression modelling.
- A study with a larger sample may have been able to identify relationships between knowledge and behaviors, and our study was not design with enough statistical power to detect significant differences.

For peer review only

- The findings of our study may not be generalizable beyond Senegal, or more specifically Senegalese adults.

For peer review only

Introduction

COVID-19 is an ongoing threat to global public health since its emergence at the end of 2019, yet little is known about how populations in low-resource settings have responded to COVID-19 in their daily lives. Although research is emerging about COVID-19 infection, strategies for prevention, and risk factors for severe disease and mortality, there is a dearth of literature about COVID-19 beliefs, specifically in the West Africa region. For example, between January and September of 2020, just 4.3% of research articles about COVID-19 were focused on Africa or a specific African nation.[1] Given that ongoing prevention strategies implemented at the country, community, and individual level will be needed to curb the COVID-19 pandemic, it is critical to study health beliefs and adherence to prevention behaviors within specific social and political contexts.[2]

Providing relevant and timely research is essential for guiding evidence-based outreach and interventions, especially during public health emergencies. It is particularly important to advance equity within public health, and the health of populations, by understanding the social contexts and health beliefs among indigenous, marginalized, and vulnerable populations around the globe.[3,4] Francophone West Africa, in particular, is underrepresented in global health research because of language barriers to publication and collaboration, leading to more research in English-speaking nations like Nigeria and Ghana.[5–7]

In the context of COVID-19, researchers must learn to apply traditional research methodologies online, such as on social media, to shed light on the etiology of COVID-19 in vulnerable and marginalized populations.[8,9] Such work creates evidence that

For peer review only

may not only be applied to other geographies but also enhance representation in research, which is crucial to creating tailored and effective interventions.[10] More broadly, research that creates evidence and informs policy about health issues like COVID-19 in West Africa and other parts of the developing world promotes global health equity and justice.[11–13] The current study provides timely, critical, and actionable information about COVID-19 beliefs and prevention behaviors for stakeholders, policy makers, and public health programs to reduce the burden of COVID-19 in West Africa by reporting on findings among a sample of adults from the West African nation of Senegal.

Senegal as a Case Study of COVID-19 Beliefs and Behaviors in West Africa

Senegal is the former capital of French West Africa and home to over 16 million people, and therefore may serve as a relevant case study for exploring COVID-19 beliefs in West Africa. Furthermore, Senegal's communication regarding COVID-19 has widely been considered successful and may have contributed to unexpectedly fewer COVID-19 cases and deaths.[14,15]. Compared to the burden of COVID-19 in Europe, the Americas, the Middle East and South Asia, the burden of COVID-19 in Africa has been quite low. Relative to the rest of the world, Africa defied initial expectations by experiencing the lowest burden of reported COVID-19 cases and deaths.[16,17] Questions remain about why COVID-19 infections are reportedly low in Africa, in particular in West Africa where there have been 377 cases per 100,000 persons cumulatively. Although a 2020 analysis by Aduh and colleagues reported on perceptions and behaviors towards COVID-19 across Sub-Saharan Africa, we have

For peer review only

much to learn from populations in densely populated West Africa, a comparatively more urban Sub-Saharan region home to more than 400 million people.[18,19]

Some researchers have questioned the accuracy of African COVID-19 case numbers,[16] hypothesizing that the burden of disease could be much higher than reported. Uncertainty about the true trajectory of COVID-19 in Africa may leave all continents vulnerable to future outbreaks, particularly regions with low herd immunity due to lack of exposure and vaccine resource constraints. It is likely that places that have not yet needed to adopt strong prevention efforts because of reportedly low case numbers therefore may be especially vulnerable to future outbreaks. Thus, despite relatively low case numbers, it is still vital to investigate COVID-19 health beliefs and prevention behaviors in West Africa peoples to identify potential correlates and predictors of viral prevention behaviors. Our study aims to contribute evidence towards this gap in the literature by adapting pre-existing research cohorts and developing novel data collection instruments to capture information about COVID-19.

Study Objectives and Research Questions

To explore COVID-19 beliefs and prevention behaviors in francophone West Africa, the current study analyzed cross-sectional survey data collected from a sample of adults in Senegal. The survey, which was administered both online and via telephone, included a module on COVID-19 behaviors and beliefs. We sought to identify correlates between respondent demographic attributes and COVID-19 beliefs and behaviors. Our investigation was theoretically informed from Rosenstock's Health Belief Model (HBM),[20] a behavior change framework for public health practice positing that knowledge of a potential negative outcome and perceived threat of that outcome

For peer review only

precede behavior change.[21] Other recent studies have similarly relied on HBM constructs to explore COVID-19. Specifically, this study applies HBM constructs to answer the following questions:

1) In Senegal, what is the level of knowledge and awareness about COVID-19?

2) To what extent is COVID-19 perceived as a health threat?

3) What is the relationship between COVID-19 beliefs and prevention behaviors?

Methods

Study Design, Participants and Setting

From June-August 2020, we conducted a multi-modal cross-sectional survey that included questions regarding COVID-19 beliefs and prevention behaviors among a convenience sample of 1,452 adults in Senegal. All respondents were participating in a separate ongoing longitudinal cohort study. Potential participants were informed about the study and provided consent to participate in the survey (online) or interview (telephone). For both online and telephone participants, all individuals were required to be 18 years or older.

All online participants were part of a cohort study about entertainment education in West Africa that began in late summer 2019; of the 1,286 cohort participants recruited online in summer 2019, 231 both were eligible to participate (i.e., Senegalese adults) and completed the COVID-19 questionnaire items. Online participants from Senegal were recruited using social media advertisements on Facebook and YouTube.

The telephone survey used a random selection of phone numbers from a pre-existing research cohort of individuals who lived in Senegal and agreed to participate in

For peer review only

media-related research and subsequent follow up surveys. COVID-19 questionnaire items were included as an accompanying section with the primary study's survey. A quota system was used to match the overall population of Senegal on sex and have an even distribution of age and level of education. A total of 1,221 participants were enrolled in the study via telephone, split approximately equally between participants living insider versus outside of Dakar, Senegal and by gender.

Data Collection

This survey used two modes of data collection: online and telephone. We opted to collect data about COVID-19 from pre-existing cohorts of participants in Senegal so as to be responsive to the ongoing and evolving global epidemic and to address evidence gaps mentioned above (see Introduction). Through our multi-modal strategy, responses about COVID-19 were collected from 1,452 Senegalese adults. Consenting participants were compensated with 2000 Central African CFA franc (~4 USD) for their time. Telephone surveys were collected by OMedia, a Dakar-based third party research firm, and overseen by researchers from UCLA (MB, DG). Online surveys were collected via Qualtrics by researchers from Drexel University (MK, PM). Incomplete responses with missing data were excluded. All study procedures and protocols were reviewed by and approved or determined to be exempt by institutional ethical review boards (see Research Ethics Approval). Figure 1 presents a flow diagram of our study recruitment and sampling.

Measurement

Survey items included a series of self-reported demographic questions (e.g., age, education) as well as a 15-item section specifically about COVID-19. Using Cronbach's

For peer review only

α, we assessed internal consistency (i.e., scale reliability) for all 15 COVID-19 survey (α= 0.7; acceptable reliability). In light of the novel nature of our data collection instrument, we determined the scale to be acceptably reliable.[22] The COVID-19 section contained three measurement domains: 1) knowledge & awareness, 2) perceived threat to health, and 3) prevention behaviors. COVID-19 items were developed based on available guidance from the WHO and CDC and were reviewed by French, English, and Wolof speaking team members. The survey was translated from English to French by study team members in Senegal (MF) and UCLA (RN).

Response options were binary (e.g., agree/disagree) or Likert-style (i.e., ordinal). COVID-19 response items were aggregated into distinct scales based on the three measurement domains. Each scale ranged from 0 to 15. As the COVID-19 items were novel, these “scales” were used as a proxy measure for determining the degree of knowledge and awareness, perceived risk, and practicing of known prevention behaviors including 1) handwashing, 2) using hand sanitizer, 3) wearing a mask, 4) social distancing, and 5) staying at home. Perceived COVID-19 risk items drew from the Health Belief Model including perceived susceptibility (“Are you afraid of catching COVID-19?”; “Do you think the COVID-19 epidemic poses a risk to Africans?”), perceived severity (“Do you think COVID-19 represents a danger to your health?”; “How stressed do you feel about hearing about COVID-19?”), and perceived behavioral control (“If you thought you had caught COVID-19, would you know who to contact?”).

Data Analysis

Descriptive statistics were generated for demographic attributes and COVID-19 items. Chi-squared tests assessed differences in demographic attributes between the

For peer review only

online and telephonic collection methods. Significance was determined at the alpha=0.05 level. For multivariate analysis, we recoded level of education, marital status, location (e.g., urban/rural), religion, and occupation to reduce the number of categories, creating binary or ordinal variables, as shown in Table 1. Participant age was collected as a continuous measure and recoded as both quartile age groups and as binary (above versus below median age). Median values were calculated for each of the three COVID-19 scales and used as thresholds to create binary variables (e.g., 0=low knowledge;1=high knowledge).

We built five logistic regression models that predicted COVID-19 behaviors individually (i.e., hand washing, mask wearing, social distancing, staying home) and in aggregate, the results of which are presented as Forest plots (Figure 2). For individual behavior models, dependent variables were whether or not participants practiced prevention behaviors daily in the past week; for the aggregate model the dependent variable was the prevention behavior scale ranging from 0 (practice no behaviors in past week) to 15 (practice all behaviors daily). Independent variables in each model included demographic items, perceived threat (high/low), and knowledge & awareness (high/low). All statistical analyses were conducted using Stata IC software (version 15). Forest plots were generated using the 'forestplot' package in RStudio software.[23]

Patient and Public Involvement

This study is a cross-sectional analysis of surveys conducted in 2020 using a multi-modal recruitment strategy. No patients were involved in our study. All analyses were conducted in collaboration with research partners in Dakar, Senegal so it was not

For peer review only

appropriate to involve specific public communities or patients or in the design, conduct, reporting or dissemination plans.

Results

A total of 1,452 Senegalese adults participated in our cross-sectional survey about COVID-19. Table 1 presents demographics characteristics for the study sample. Table 2 presents univariate descriptive statistics for COVID-19 survey items. Table 3 presents unadjusted regression models and Table 4 presents adjusted regression models. Figure 2 presents a Forest plot of our logistic regression model predicting prevention behaviors.

Sample Characteristics

Most participants identified as Muslim (94.5%; n=1,372) and primarily lived in in an urban area (77.1%; n=1,118). Nearly half completed secondary education (i.e., high school or more; 49.9%; n=725). Most participants were single (55.4%; n=804). Participants' ages ranges from 18-44 years, with a median age of 26 years and mean of 28.0 years (SD=7.6; see Supplemental Information File). The sample was evenly split between females and males (50.1% versus 49.9%). More than a third of participants worked in the informal sector (e.g., craftsperson, restauranteur; 39.2%; n=569) and another third were currently students (27.6%; n=400). Most participants completed the survey via phone rather than online, respectively 84.1% and 15.9%.

We identified some differences between our two recruitment methods – i.e., online versus telephone (see Supplemental Information File). Compared to participants recruited online, participants recruited telephonically were more likely to be older on

For peer review only

average (telephone=28.5 years versus online=25.0; p<.001), live in rural versus urban settings (23.0% versus 4.4%; p<.001), and have less than college education (16.1% versus 79.7%; p<.001). Half of telephone participants were male, compared to less than four in ten online participants (49.9% versus 39.4%; p=.003). Significant differences were also identified between recruitment methods for relationship status (p<.001), profession (p<.001), and religion (p<.001). Compared to telephone participants, online participants also had significantly higher COVID-19 knowledge and awareness (p<.001) and were significantly more likely to engage in prevention behaviors (p<.001).

Knowledge and Awareness

As shown in Table 2, of the 1,452 participants in our study sample less than 1% had not heard about COVID-19 (0.4%; n=6). More than nine in ten participants knew that hand washing (94.8%; 1,377) and mask wearing in public (97.8%; n=1,420) were effective methods to prevent COVID-19 transmission and/or infection. About half were aware that staying at home (48.6%; n=705) or avoiding groups of more than ten people (50.8%; n=738) were COVID-19 prevention recommendations. Nearly four in five participants (79.4%; n=1,149) said that they would know who to contact (e.g., testing, healthcare) if they suspected themselves to have had COVID-19. The median knowledge score was 12.5 out of 15 (range=0 to 15; mean=11.8 / SD=2.6; see Supplemental Information File).

Perceived Threat

As shown in Table 2, nine in ten participants (94.6%; n=1,369) perceived COVID-19 to be a very or somewhat serious danger to their health. Almost three-quarters (73.3%; n=1,061) said that COVID-19 posed a serious risk to Africans. Median

For peer review only - http://bmjopen.bmj.com/site/about/guidelines.xhtml

perceived threat score was 11.6 out of 15 (mean=10.9 /SD=3.5; see Supplemental Information File).

Prevention Behaviors

As shown in Table 2, nine in ten participants reported washing their hands multiple times per day (94.4%; n=1,365). Nearly eight in ten used hand sanitizer multiple times per day (77.4%; n=1,123). Approximately seven in ten participants wore masks every time they left their home (65.6%; n=951). Six in ten reported practicing social distancing all or most of the time (59.3%; n=1,064). Half of participants reported staying home all or most of the time (52.3%; n=758). Less than one in ten people went out as usual (6.2%; n=90) or ignored social distancing (5.7%; n=83). Less than one in one hundred participants never wore a mask in public (0.4%; n=6). The median prevention behaviors score was 11 out of 15 (mean=10.5/ SD=2.3; see Supplemental Information File). Unadjusted bivariate regression models for predictors of prevention behaviors are presented in Table 3 for reference. Below we discuss results for adjusted regression models shown in Table 4 and Figure 2.

Regression Analysis COVID-19 behaviors were significantly associated with COVID-19 beliefs and participant demographic attributes (see Figure 2, Table 4). We also observed differences in behaviors based on our participant recruitment method. Compared to participants recruited telephonically, participants recruited online were significantly more likely to socially distance (OR=3.33; p=<.001), wear masks (OR=3.26; p=.007), and stay home (OR=1.63; p=.013).

Participants who reported high degrees of perceived threat about COVID-19 were significantly more likely to practice prevention behaviors in both the aggregate and

For peer review only

individual models (OR=1.69; *p*<.001). Knowledge and awareness related to COVID-19 poorly predicted practicing prevention behaviors, although high levels of knowledge and awareness were positively associated with participants' use of hand sanitizer (OR=1.47; *p*=.035). At the same time, higher levels of knowledge and awareness about COVID-19 was negatively associated with staying home (OR=0.64; *p*=.002).

Compared to participants with less than a high school degree, participants with high school education or above were more likely to stay home (OR=1.67; *p*<.001), practice social distancing (OR=1.28; *p*=.070), and use hand sanitizer (OR=1.35; *p*=.062). Compared to urban participants, rural participants were more likely to socially distance (OR=1.36; *p*=.032), stay home (OR=1.80; *p*<.001), and wear a mask (OR=2.17; *p*=.021), but less likely to user hand sanitizer (OR=0.67; *p*=.010). Use of hand sanitizer was more likely among formal workers compared to students (OR=2.18; *p*=.002). Compared to participants less than 26 years old, older participants (over 26) were significantly less likely to stay home (OR=0.76; *p*=.045).

Sex-based differences were observed for COVID-19 prevention behaviors. Males had 41% lower odds of practicing *all five* COVID-19 prevention behaviors compared to females (OR=0.59; *p*<.001). Males also reported lower adherence to COVID-19 prevention measures for each of the five behaviors individually. Compared to females, males were 61% less likely to wash their hands on a daily basis (*p*<.001), 51% less likely to report staying home (*p*<.001), 39% less likely to wear masks daily (*p*<.037), 38% less likely to practice social distancing (*p*<.001), and 37% less likely to use hand sanitizer (*p*=.001).

For peer review only

For peer review only

Table 1. Sample characteristics. N=1,452. Data collected via phone and online surveys from Senegalese adults between June-August 2020.

**Characteristic**	**Category**	**#**	**%**
Survey modality	Telephone	1,221	84.1
	Online	231	15.9
Age Quartile	<21	353	24.3
	22-25	352	24.2
	26-34	354	24.4
	35+	393	27.1
Sex	Female	752	51.8
	Male	700	48.2
Location	Urban	1,118	77.1
	Rural	291	20.1
	Urban + Rural	42	2.9
Education	Superior	381	26.2
	Secondary	344	23.7
	Primary	284	19.6
	Middle	231	15.9
	No	145	10.0
	Other	67	4.6
Marital status	Single	804	55.4
	Married - monogamous	460	31.7
	Married - polygamist	161	11.1
	Divorced	20	1.4
	Widower	7	0.5
Occupation	Informal sector	569	39.2
	Student	400	27.6
	Formal sector	225	15.5
	Other	112	7.7
	Housewife	96	6.6
	Retired, out of work	49	3.4
	Religious leader	1	0.1
Religion	Muslim	1,372	94.5
	Catholic	65	4.5
	Protestant / Evangelical	8	0.6
	No	3	0.2
	Other	3	0.2
	Traditionalist	1	0.1

For peer review only

**Table 2.** Frequency (#) and percent (%) for responses to COVID-19 questionnaire items. N=1,452 Senegalese adult participants. All responses collected between June-August 2020.		
	**#**	**%**
**Knowledge and Awareness Items**		
Have you ever heard of COVID-19, also called Coronavirus?		
Yes	1,446	99.6
No	6	0.4
What protective measure against COVID-19 / coronavirus do you know?		
Stay at home	705	48.6
Wash your hands regularly	1,377	94.8
Wear a mask in public	1,420	97.8
Avoid meeting with groups of more than 10 people	738	50.8
**Prevention Behavior Items (Past Week)**		
Staying at home		
Partly (I try to limit time away from home but go out several times a week)	602	41.5
Most of the time (I go out about once a week)	476	32.8
All the time (I didn't leave the house)	282	19.5
I went out as much as usual	90	6.2
Wash your hands with soap and water		
Several times a day, even if they do not seem dirty	830	57.4
Every day, as soon as they are dirty after the toilet, the kitchen or the work	535	37.0
Very rarely / Never	44	3.0
Once a day approximately	38	2.6
Wear a mask when you leave your home		
Each time	951	65.6
Most of the time	403	27.8
Sometimes	90	6.2
Never / I don't have a mask	6	0.4
Use hand sanitizer gel		
Every day, but only in the places where it is needed	595	41.0
Several times a day	528	36.4
Rarely / never	215	14.8
About once a day	112	7.7
Practice social distancing (limit the number of other people)		
Most of the time (I go out rarely and keep a distance)	557	38.4
Partly (I don't limit my social interactions but I keep my distance)	507	35.0
All the time (I am confined to my home)	303	20.9
Never (I act as usual: I pass other people and I shake their hands)	83	5.7
**Perceived Threat**		
Do you think COVID-19 represents a danger to your health?		
Yes, very serious	1,152	79.6
Somewhat serious	217	15.0

For peer review only

No	78	5.4
Are you afraid of catching COVID-19?		
Very afraid	913	63.1
Relatively afraid	347	24.0
Not afraid	186	12.9
Do you think the COVID-19 epidemic poses a risk to Africans?		
Yes, a serious risk	1,061	73.3
Yes, but a reasonable risk	283	19.5
No, a low risk	104	7.2
If you thought you had caught COVID-19, would you know who to contact?		
Yes	1,149	79.4
No	298	20.6
How stressed do you feel about hearing about COVID-19?		
Somewhat stressed	586	40.5
Stressed a little	366	25.3
Stressed a lot	262	18.1
Not at all stressed	234	16.2

For peer review only

Table 3. Univariate logistic regression models predicting COVID-19 prevention behaviors (aggregate + individually). N=1,452. All estimates presented are not adjusted for other model variables. All responses collected between June-August 2020. Alpha = 0.05. OR = odds ratio.

	**All Behaviors**				**Using Hand Sanitizer**				**Social Distancing**			
Predictor	OR	95% CI		*p*	OR	95% CI		*p*	OR	95% CI		*p*
Online participant[1]	2.39	1.80	3.18	<.001	2.46	1.61	3.75	<.001	3.33	2.36	4.70	<.001
COVID Perceived threat	1.71	1.37	2.13	<.001	1.75	1.35	2.28	<.001	2.01	1.62	2.51	<.001
Rural[2]	1.25	0.96	1.63	0.104	0.56	0.42	0.75	<.001	1.14	0.87	1.48	0.334
High school or above[3]	0.76	0.61	0.95	<.001	0.78	0.59	1.01	<.01	0.70	0.56	0.87	<.001
Formal worker[4]	0.98	0.73	1.33	0.018	1.49	1.00	2.22	0.059	1.10	0.81	1.50	0.002
Student[4]	1.56	1.26	1.94	0.901	1.54	1.20	1.98	0.053	1.67	1.35	2.06	0.532
COVID KAP	1.47	1.16	1.86	<.01	2.13	1.56	2.90	<.001	1.55	1.22	1.97	<.001
Single[5]	0.92	0.74	1.14	0.446	1.07	0.83	1.37	0.607	0.96	0.78	1.19	0.722
26 years or older[6]	0.80	0.64	0.99	0.044	1.09	0.85	1.39	0.511	0.81	0.66	1.00	0.048
Male[6]	0.49	0.39	0.61	<.001	0.60	0.47	0.77	<.001	0.52	0.42	0.64	<.001

	**Staying Home**				**Hand Washing**				**Mask Wearing**			
Predictor	OR	95% CI		*p*	OR	95% CI		*p*	OR	95% CI		*p*
Online participant[1]	1.63	1.22	2.17	<.01	2.49	1.07	5.78	0.029	2.17	1.04	4.53	0.036
COVID Perceived threat	1.63	1.32	2.01	<.001	3.86	2.11	7.05	<.001	3.16	1.87	5.34	<.001
Rural[2]	1.65	1.27	2.15	<.001	1.35	0.74	2.48	0.329	2.01	1.06	3.82	0.030
High school or above[3]	0.83	0.67	1.03	<.001	1.06	0.66	1.68	0.640	1.40	0.94	2.07	0.517
Formal worker[4]	0.87	0.65	1.17	0.093	1.48	0.73	3.00	0.814	1.93	1.05	3.56	0.094
Student[4]	1.71	1.39	2.11	0.348	0.90	0.58	1.40	0.273	0.87	0.58	1.32	0.035
COVID KAP	0.91	0.73	1.15	0.439	2.41	1.29	4.49	0.005	1.55	0.93	2.56	0.090
Single[5]	1.00	0.81	1.23	0.985	0.59	0.37	0.95	0.028	0.54	0.35	0.85	0.006
26 years or older[6]	0.74	0.60	0.91	0.005	1.53	0.98	2.41	0.063	1.39	0.92	2.11	0.119
Male[6]	0.45	0.37	0.56	<.001	0.34	0.21	0.56	<.001	0.54	0.35	0.82	0.004

[1]Ref=Telephone participant; [2]Ref=Urban; [3]Ref=Below high school; [4]Ref=Informal worker, housewife, retired, out of work, religious leader, other; [5]Ref=Married (monogamous + polygamous), divorced, widower; [6]Ref=Female

For peer review only - http://bmjopen.bmj.com/site/about/guidelines.xhtml

Table 4. Multivariate logistic regression models predicting COVID-19 prevention behaviors (aggregate + individually). N=1,452. All estimates presented are adjusted for other model variables. All responses collected between June-August 2020. Alpha = 0.05. aOR = adjusted odds ratio.

	All Behaviors				Using Hand Sanitizer				Social Distancing			
Predictor	aOR	95% CI		*p*	aOR	95% CI		*p*	aOR	95% CI		*p*
Online participant[1]	1.93	1.29	2.87	0.001	1.62	0.94	2.78	0.079	3.20	2.05	5.00	<.001
COVID Perceived threat	1.69	1.33	2.13	<.001	1.69	1.28	2.22	<.001	2.03	1.61	2.57	<.001
Rural[2]	1.49	1.12	1.98	0.006	0.67	0.50	0.91	0.010	1.36	1.03	1.81	0.032
High school or above[3]	1.33	1.01	1.76	0.044	1.35	0.98	1.84	0.062	1.28	0.98	1.67	0.070
Formal worker[4]	1.28	0.86	1.90	0.230	2.18	1.32	3.60	0.002	1.42	0.95	2.13	0.090
Student[4]	1.07	0.76	1.52	0.691	1.33	0.90	1.97	0.149	0.98	0.70	1.38	0.927
COVID KAP	0.96	0.72	1.29	0.798	1.47	1.03	2.10	0.035	0.89	0.66	1.18	0.411
Single[5]	0.75	0.56	1.02	0.070	1.20	0.86	1.69	0.281	0.79	0.59	1.06	0.116
26 years or older[6]	0.75	0.56	1.00	0.054	1.19	0.86	1.64	0.287	0.78	0.59	1.03	0.077
Male[6]	0.59	0.46	0.75	<.001	0.64	0.49	0.84	0.001	0.62	0.49	0.79	<.001
	**Staying Home**				**Hand Washing**				**Mask Wearing**			
Predictor	aOR	95% CI		*p*	aOR	95% CI		*p*	aOR	95% CI		*p*
Online participant[1]	1.62	1.09	2.42	0.018	2.48	0.89	6.96	0.084	3.25	1.31	8.04	0.011
COVID Perceived threat	1.62	1.29	2.03	<.001	3.09	1.67	5.71	<.001	2.81	1.64	4.82	<.001
Rural[2]	1.80	1.35	2.38	<.001	1.52	0.81	2.86	0.189	2.17	1.12	4.19	0.021
High school or above[3]	1.67	1.28	2.17	<.001	0.85	0.49	1.45	0.546	0.82	0.50	1.36	0.448
Formal worker[4]	0.98	0.67	1.44	0.918	1.54	0.67	3.53	0.311	2.44	1.03	5.75	0.042
Student[4]	0.89	0.64	1.25	0.510	1.12	0.58	2.16	0.733	1.10	0.60	1.99	0.760
COVID KAP	0.64	0.48	0.85	0.002	1.78	0.88	3.63	0.110	1.14	0.64	2.04	0.654
Single[5]	0.85	0.64	1.13	0.260	0.93	0.50	1.71	0.809	0.73	0.41	1.29	0.277
26 years or older[6]	0.76	0.58	0.99	0.045	1.40	0.80	2.46	0.243	1.05	0.63	1.77	0.847
Male[6]	0.49	0.39	0.62	<.001	0.39	0.23	0.66	<.001	0.61	0.39	0.97	0.037

[1]Ref=Telephone participant; [2]Ref=Urban; [3]Ref=Below high school; [4]Ref=Informal worker, housewife, retired, out of work, religious leader, other; [5]Ref=Married (monogamous + polygamous), divorced, widower; [6]Ref=Female

For peer review only - http://bmjopen.bmj.com/site/about/guidelines.xhtml

Discussion

In our cross-sectional sample of Senegalese adults, we observed that the majority of participants practiced COVID-19 prevention behaviors on a daily basis. Mask wearing, hand washing, and use of hand sanitizer were most frequently reported. Social distancing and staying at home were also reported albeit to a lower degree. We also identified a range of psychosocial and demographic predictors for COVID-19 prevention behaviors. For example, participants who perceived COVID-19 to be a threat were significantly more likely to practice every prevention behavior measured than those who did not perceive COVID-19 to be a threat. This is in line with existing health behavior research regarding perceived threat as a necessary precursor to behavior change.[20,21] Our findings from Senegal not only contribute to a growing body of literature about COVID-19 beliefs and behaviors in developing African nations, but also support prior research in both developing and developed nations about predictors of health behaviors.

Prevention Behaviors

Males were less likely than females to report practicing any of the prevention behaviors that we studied. In the US, a developed nation, previous research shows that females are more likely to engage generally in preventive health behaviors adhering to public health recommendations, such as cancer screenings, exercise, and dietary intake.[24,25] Specific to COVID-19, females have also been shown to more frequently practice prevention behaviors like mask wearing, and this is true both in developed nations like the US,[26,27] as well as in developing nations like South Africa, [28] Chile,[29] Turkey,[30], and China.[31] However, studies in Sierra Leone and Cameroon

For peer review only

have both found that females were less likely to engage in COVID-19 prevention behaviors, contradictory to our findings from Senegal. In general, males may also be more likely to engage in risk taking behaviors,[32] whereas females are more risk averse.[33] Future research into adherence of recommended COVID-19 prevention behaviors should explore sex-based differences in risk perceptions and tolerance.

Practicing COVID-19 prevention behaviors was also positively associated with living in a rural versus urban area. It is possible that people residing in rural areas have an easier time implementing preventive behaviors such as staying at home and social distancing than people in more densely populated urban areas. Finally, Participants with at least a high school education were also more likely to engage in preventive behaviors. Educational differences in adoption of COVID-19 preventive health behaviors has previously been reported in studies in other nations like South Africa and China.[31,34,35] Such demographic variations in practicing COVID-19 prevention behaviors indicate priority populations for future health communication campaigns. For example, people in urban areas may require more prompting and resources to implement preventive behaviors in the future.

Considering our multi-modal recruitment strategy, we found that respondents from the online sample demonstrated greater knowledge and awareness of COVID-19 and engaged in prevention behaviors more often compared to respondents in the telephone sample, suggesting that strategies leveraging online communications and communities of users will be important to continue supporting and promoting prevention behaviors, in particular to build vaccine confidence as vaccine availability increases throughout the region. Differences between respondents' based on recruitment method

For peer review only

could indicate similar differences in Senegalese adults in general depending on whether they had access to and were frequent users of the internet. This provides support for both digital and on-the-ground messaging and communication to strengthen pro-prevention beliefs, attitudes, and behaviors. For example, health promotion campaigns could engage local social media influencers or bloggers to promote positive attitudes towards COVID-19 prevention behaviors, including vaccination, and rely on their networks of users to further disseminate health communications via word of mouth to their families and friends online and offline.

Health Beliefs

Our study findings comport with a robust body of evidence about COVID-19 knowledge, awareness, attitudes, and practices. In Senegal, we observed that knowledge, perceived risk, and practicing prevention behaviors for COVID-19 were very high in general – albeit with some demographic variations. A 2020 international review by Puspitasari and colleagues reported generally high knowledge and awareness about COVID-19, pro-prevention attitudes, and high adherence to COVID-19 prevention behaviors like handwashing and social distancing.[36] Closer to the West African and Senegalese contexts, Aduh and colleagues reported that in sub-Saharan Africa moderate to high levels of COVID-19 knowledge and perceived risk.[18]

We did not find an association between knowledge and engaging in prevention behaviors, a finding both contrary to and in line with prior international research of infectious disease practices in developing nations. For example, a 2020 study in Guyana found that knowledge was a significant predictor of practicing malaria and dengue prevention behaviors but not for Zika virus and leishmaniosis, a common

For peer review only

protozoan-caused skin infection.[37] Specific to COVID-19, a 2020 study in China reported that participants with increased knowledge were more likely to go to crowded places and less likely to wear masks.[31] Finally, a 2021 scoping review of 28 studies on COVID-19 beliefs and preventive practices in 9 sub-Saharan African nations – not including Senegal – reported that although knowledge about COVID-19 was generally high, it was not always associated with increased risk perceptions or attitudes towards the virus, and by extension was a poor predictor of prevention behaviors.[38]

In the current study, we found that perceived COVID-19 risk was a stronger predictor of prevention behaviors, which is also supported by previous international research.[2,39] Low perceptions of risk may result in low compliance with virus restrictions and adherence to prevention behaviors,[40–42] and therefore facilitates COVID-19's spread. This confirms another recent study which recruited using social media from the United States, China, Taiwan, and Mexico.[2] Hsing and colleagues found that perceived COVID-19 severity and susceptibility – constructs that comprise perceived risk/threat in the Health Belief Model – were both predictors of hand washing and social distancing.[2] In our study based in Senegal, perceived risk not only predicted hand washing and social distancing, it also predicted staying home, using hand sanitizer, and mask wearing. These findings suggest that to prevent COVID-19 spread in Senegal and other parts of francophone West Africa, we need to focus more on perceived risk than knowledge. For example, health agencies and organizations should promote health communications that highlight Senegalese residents' susceptibility to contracting COVID-19 as well as its potential health impacts. Messaging

For peer review only - http://bmjopen.bmj.com/site/about/guidelines.xhtml

should be targeted towards groups that we identified as having lower adherence to prevention behaviors, in particular men.

A Focus on Senegal

In general, Senegalese participants had high levels of COVID-19 knowledge and awareness, perceived COVID-19 to be a threat both personally and across Senegal, and in most cases adopted known prevention behaviors on a daily or regular basis. Looking forward, as vaccines become more widely available in Senegal and many West African nations, inequities in access will make sustaining preventive behaviors paramount to continued control of the virus.[43] Our findings suggest that other nations should look to Senegalese communications about COVID-19 to learn messages that may resonate more effectively with their populations to increase prevention behaviors.

As has been stated, most participants reported moderate to high adherence to COVID-19 prevention behaviors. To explain our findings, we must consider lessons learned from previous regional epidemics and how they relate to evolving social contexts (e.g., norms, culture, population attributes), in particular Ebola.[9] Many behaviors associated with preventing COVID-19 were already familiar to residents of African nations who experienced Ebola outbreaks, including hand washing, use of personal protective equipment, and social distancing.[44] COVID-19 in West Africa comes several years after the Ebola outbreak in 2014-2015, which not only tested the limits of the health systems but also demonstrated its resilience. For example, although Senegal experiences a nationwide shortage of doctors – just 7 per 100,000 persons in 2020 – the nation has been comparatively effective at controlling COVID-19 infections. The first case of COVID-19 in Senegal, a country of 17 million, was reported in early

For peer review only - http://bmjopen.bmj.com/site/about/guidelines.xhtml

March 2020, prompting the Ministry of Health to develop protocols to monitor and address cases within Senegal and at its borders and airports. Cases in Senegal remained low throughout Spring, but like other nations rose considerably in the latter half of 2020 and first months of 2021.[45,46] On August 8th, 2020, Senegal's Minister of Interior mandated mask wearing in indoor spaces, banned gatherings in public spaces like beaches, theatres, and sporting grounds, and prohibited demonstrations on public highways.[47] These restrictions were emphasized in particular for the densely populated region of Dakar, but did not include mandatory lockdowns, local travel restrictions, or school/university closures. Although our survey was conducted in summer 2020, our findings continue to provide relevant context to current COVID-19 epidemiological data in Senegal: as of January 27, 2022, the 7-day rolling average for COVID-19 cases per million people was lower in Senegal than both the rest of Africa and the world – 7.95 versus 22.65 versus 427.1, respectively.[48]

Senegal's success at controlling the virus throughout 2020 has been attributed to many factors, including a citizenry who has witnessed the devastating impacts of the Ebola epidemic. More broadly across West Africa, citizens may be more likely to comply with public health restrictions because of their experience with Ebola,[49] and this extends to individual behavior changes as well. For example, one study reported that handwashing was shown to increase in Senegal as a result of Ebola education campaigns.[50] In our analysis, we found that daily hand washing was the prevention behavior most often reported by participants. Future health campaigns may look at Ebola prevention messages to identify behaviors and information that may be reinforced in the context of COVID-19.

For peer review only - http://bmjopen.bmj.com/site/about/guidelines.xhtml

Limitations

Our findings may not be generalizable beyond Senegal, or more specifically Senegalese adults, because of our targeted multi-modal recruitment strategy. The recruitment strategy increased the potential for selection bias because participants were recruited online and on-the-ground in Senegal; thus, all participants, by the nature of the recruitment methods, had access to the internet and/or a cell phone. We did not identify significant relationships for many factors in our regression model because our cross-sectional study was not statistically powered to detect significant differences and we did not assess the discriminatory power of our novel questionnaire items or scales. Staying at home or social distancing prevention behaviors may have been confounded by other factors not included in our regression modelling, in particular government lockdowns, local travel restrictions, school/university closures, and whether a participant worked outside of the home or not. Although our COVID-19 survey items were developed by the study team based on available WHO guidance, they were not validated tools like the ICP COVID survey and therefore have limited transferability to similar investigations.

Conclusion

Our study contributes to a growing body of literature about COVID-19 in Africa,[51,52] and is the first study to look specifically at knowledge and prevention behaviors in the francophone West African nation of Senegal. In Senegal, we observed that knowledge and perceived risk of COVID-19 were very high in general. But, risk was a stronger and more influential predictor of COVID-19 prevention behaviors. COVID-19 prevention behaviors also varied by sex, education, location, and country. These findings suggest that in West Africa, nations should seek to elevate risk perceptions

For peer review only - http://bmjopen.bmj.com/site/about/guidelines.xhtml

about COVID-19 and develop messaging to better target certain groups. Elevating the perceived risk of a population is a difficult issue because public health programs do not want to raise unnecessary alarm. Thus, messages around risk of COVID-19 should be accompanied by empowering messages of how individuals can protect themselves, their families, and their communities. Health campaigns should look backwards to successes with Ebola for improving health messaging about COVID-19, and may also look to replicate the successes of regional examples like Senegal.

Figure 1. Study recruitment and sampling diagram.

Figure 2. Forest plot diagrams for logistic regression models predicting COVID-19 prevention behaviors (aggregate + individually). Square box represents point estimate and horizontal bar represents 95% CI. Confidence intervals that cross OR threshold of 1.0 are not statistically significant at the alpha=0.05 level. All estimates presented are adjusted for other model variables. N=1,452. All responses collected between June-August 2020.

Data availability statement

No data are available.

Ethics statements

Patient consent for publication: Not required.

Research ethics approval: All study procedures and protocols were approved by institutional ethical review board at Drexel University (Protocol ID: 00015164) and

For peer review only

UCLA (Protocol ID: 16-000393). Written informed consent was obtained from all participants.

Acknowledgements

We are grateful for support from our French and Senegalese collaborators in Dakar, Senegal at The African Health and Education Network (NGO RAES) and at Université Cheikh-Anta-Diop. Data collection was completed while the first (MK) and last author (PM) were affiliated with the Drexel University Dornsife School of Public Health, and while the second author (MB) was a graduate student at UCLA Fielding School of Public Health. Finally, we are thankful to all participants in this study.

Competing Interests

None declared.

Funding

This study received funding from the Bill & Melinda Gates Foundation (OPP1181104). Disclaimer. The study sponsors had no role in study design, data collection, data analysis, our decision to publish, or preparing the manuscript.

Contributors

All authors contributed to the editing and reviewing of this paper. MK, MB, DG, and PM were involved in conceptualizing this article. MF and RN led development of the data collection tool. MK and RN designed analyses for this manuscript, and MK conducted the analyses. MK, MB, DG, and PM wrote the paper. All authors reviewed, edited, and revised the paper.

For peer review only

References

1 Naidoo AV, Hodkinson P, Lai King L, *et al.* African authorship on African papers during the COVID-19 pandemic. *BMJ Glob Health* 2021;**6**:e004612. doi:10.1136/bmjgh-2020-004612

2 Hsing JC, Ma J, Barrero-Castillero A, *et al.* Influence of Health Beliefs on Adherence to COVID-19 Preventative Practices: International, Social Media–Based Survey Study. *J Med Internet Res* 2021;**23**:e23720. doi:10.2196/23720

3 Alistair Mallard, Maria Amalia Pesantes, Carol Zavaleta-Cortijo, *et al.* An urgent call to collect data related to COVID-19 and Indigenous populations globally. *BMJ Glob Health* 2021;**6**:e004655. doi:10.1136/bmjgh-2020-004655

4 Sarah Wehbe, Fahme SA, Rizk A, *et al.* COVID-19 in the Middle East and North Africa region: an urgent call for reliable, disaggregated and openly shared data. *BMJ Glob Health* 2021;**6**:e005175. doi:10.1136/bmjgh-2021-005175

5 Bodewes AJ, Kunst AE. Involving hard-to-reach ethnic minorities in low-budget health research: lessons from a health survey among Moluccans in the Netherlands. *BMC Res Notes* 2016;**9**:319. doi:10.1186/s13104-016-2124-1

6 Dusek G, Yurova Y, P. Ruppel C. Using Social Media and Targeted Snowball Sampling to Survey a Hard-to-reach Population: A Case Study. *Int J Dr Stud* 2015;**10**:279–99. doi:10.28945/2296

7 Wilkerson JM, Iantaffi A, Grey JA, *et al.* Recommendations for Internet-Based Qualitative Health Research With Hard-to-Reach Populations. *Qual Health Res* 2014;**24**:561–74. doi:10.1177/1049732314524635

8 Ali SH, Foreman J, Capasso A, *et al.* Social media as a recruitment platform for a nationwide online survey of COVID-19 knowledge, beliefs, and practices in the United States: methodology and feasibility analysis. *BMC Med Res Methodol* 2020;**20**:116. doi:10.1186/s12874-020-01011-0

9 Bavel JJV, Baicker K, Boggio PS, *et al.* Using social and behavioural science to support COVID-19 pandemic response. *Nat Hum Behav* 2020;**4**:460–71. doi:10.1038/s41562-020-0884-z

10 Redwood S, Gill PS. Under-representation of minority ethnic groups in research — call for action. *Br J Gen Pract* 2013;**63**:342–3. doi:10.3399/bjgp13X668456

11 Braveman P, Gruskin S. Defining equity in health. *J Epidemiol Community Health* 2003;**57**:254–8. doi:10.1136/jech.57.4.254

For peer review only

12 Cacari-Stone L, Wallerstein N, Garcia AP, *et al.* The Promise of Community-Based Participatory Research for Health Equity: A Conceptual Model for Bridging Evidence With Policy. *Am J Public Health* 2014;**104**:1615–23. doi:10.2105/AJPH.2014.301961

13 Östlin P, Schrecker T, Sadana R, *et al.* Priorities for Research on Equity and Health: Towards an Equity-Focused Health Research Agenda. *PLOS Med* 2011;**8**:e1001115. doi:10.1371/journal.pmed.1001115

14 BBC News. Coronavirus in Senegal: Keeping Covid-19 at bay. BBC News. 2020.https://www.bbc.com/news/world-africa-54388340 (accessed 30 Mar 2021).

15 FP Analytics. The COVID-19 Global Response Index: A country-by-country assessment of government responses to the pandemic. Foreign Policy. 2021.https://globalresponseindex.foreignpolicy.com/ (accessed 30 Mar 2021).

16 Chitungo I, Dzobo M, Hlongwa M, *et al.* COVID-19: Unpacking the low number of cases in Africa. *Public Health Pract* 2020;**1**:100038. doi:10.1016/j.puhip.2020.100038

17 Mbow M, Lell B, Jochems SP, *et al.* COVID-19 in Africa: Dampening the storm? *Science* 2020;**369**:624–6. doi:10.1126/science.abd3902

18 Aduh U, Folayan MO, Afe A, *et al.* Risk perception, public health interventions, and Covid-19 pandemic control in sub-saharan Africa. *J Public Health Afr* Published Online First: 9 October 2020. doi:10.4081/jphia.2021.1622

19 Worldometer. Realtime Tracker: Population of Western Africa. 2021.https://www.worldometers.info/world-population/western-africa-population/ (accessed 22 Apr 2021).

20 Rosenstock IM. Historical Origins of the Health Belief Model. *Health Educ Monogr* 1974;**2**:328–35. doi:10.1177/109019817400200403

21 Glanz K, Rimer BK, Viswanath K. *Health Behavior: Theory, Research, and Practice*. John Wiley & Sons 2015.

22 DeVillis RF. Scale development: Theory and applications. 1991.

23 Gordon M, Lumley T, Gordon MM. Package 'forestplot'. *Adv For Plot Using 'grid'Graphics* 2016.

24 Berrigan D, Dodd K, Troiano RP, *et al.* Patterns of health behavior in U.S. adults. *Prev Med* 2003;**36**:615–23. doi:10.1016/S0091-7435(02)00067-1

25 Liang W, Shediac-Rizkallah MC, Celentano DD, *et al.* A population-based study of age and gender differences in patterns of health-related behaviors. *Am J Prev Med* 1999;**17**:8–17. doi:10.1016/S0749-3797(99)00040-9

For peer review only

26 Naeim A, Baxter-King R, Wenger N, *et al.* Effects of Age, Gender, Health Status, and Political Party on COVID-19–Related Concerns and Prevention Behaviors: Results of a Large, Longitudinal Cross-sectional Survey. *JMIR Public Health Surveill* 2021;**7**:e24277. doi:10.2196/24277

27 Okten IO, Gollwitzer A, Oettingen G. Gender differences in preventing the spread of coronavirus. *Behav Sci Policy* 2020;**6**:14.

28 Majam M, Fischer A, Phiri J, *et al.* International citizen project to assess early stage adherence to public health measures for COVID-19 in South Africa. *PLOS ONE* 2021;**16**:e0248055. doi:10.1371/journal.pone.0248055

29 Bronfman N, Repetto P, Cordón P, *et al.* Gender Differences on Psychosocial Factors Affecting COVID-19 Preventive Behaviors. *Sustainability* 2021;**13**:6148. doi:10.3390/su13116148

30 Yıldırım M, Geçer E, Akgül Ö. The impacts of vulnerability, perceived risk, and fear on preventive behaviours against COVID-19. *Psychol Health Med* 2021;**26**:35–43. doi:10.1080/13548506.2020.1776891

31 Zhong B-L, Luo W, Li H-M, *et al.* Knowledge, attitudes, and practices towards COVID-19 among Chinese residents during the rapid rise period of the COVID-19 outbreak: a quick online cross-sectional survey. *Int J Biol Sci* 2020;**16**:1745–52. doi:10.7150/ijbs.45221

32 Byrnes JP, Miller DC, Schafer WD. Gender differences in risk taking: A meta-analysis. *Psychol Bull* 1999;**125**:367–83. doi:10.1037/0033-2909.125.3.367

33 Adhikari B, O'Leary VE. Gender Differences in Risk Aversion: A Developing Nation's Case. Rochester, NY: : Social Science Research Network 2011. https://papers.ssrn.com/abstract=1522974 (accessed 9 Sep 2021).

34 Chen X, Chen H. Differences in Preventive Behaviors of COVID-19 between Urban and Rural Residents: Lessons Learned from A Cross-Sectional Study in China. *Int J Environ Res Public Health* 2020;**17**:4437. doi:10.3390/ijerph17124437

35 Kollamparambil U, Oyenubi A. Behavioural response to the Covid-19 pandemic in South Africa. *PLOS ONE* 2021;**16**:e0250269. doi:10.1371/journal.pone.0250269

36 Puspitasari IM, Yusuf L, Sinuraya RK, *et al.* Knowledge, Attitude, and Practice During the COVID-19 Pandemic: A Review. J. Multidiscip. Healthc. 2020;**13**:727–33. doi:10.2147/JMDH.S265527

37 Aerts C, Revilla M, Duval L, *et al.* Understanding the role of disease knowledge and risk perception in shaping preventive behavior for selected vector-borne diseases in Guyana. *PLoS Negl Trop Dis* 2020;**14**:e0008149. doi:10.1371/journal.pntd.0008149

For peer review only

38 Nwagbara UI, Osual EC, Chireshe R, *et al.* Knowledge, attitude, perception, and preventative practices towards COVID-19 in sub-Saharan Africa: A scoping review. *PLOS ONE* 2021;**16**:e0249853. doi:10.1371/journal.pone.0249853

39 Li S, Feng B, Liao W, *et al.* Internet Use, Risk Awareness, and Demographic Characteristics Associated With Engagement in Preventive Behaviors and Testing: Cross-Sectional Survey on COVID-19 in the United States. *J Med Internet Res* 2020;**22**:e19782. doi:10.2196/19782

40 Chertok IRA. Perceived risk of infection and smoking behavior change during COVID-19 in Ohio. *Public Health Nurs* 2020;**n/a**. doi:10.1111/phn.12814

41 Gierlach E, Belsher BE, Beutler LE. Cross-cultural differences in risk perceptions of disasters. *Risk Anal Off Publ Soc Risk Anal* 2010;**30**:1539–49. doi:10.1111/j.1539-6924.2010.01451.x

42 Slovic P, Fischhoff B, Lichtenstein S. Why Study Risk Perception? *Risk Anal* 1982;**2**:83–93. doi:10.1111/j.1539-6924.1982.tb01369.x

43 Reuters. 'Immense joy' as Senegal begins public vaccine distribution. Reuters. 2021.https://www.reuters.com/article/us-health-coronavirus-senegal-vaccinatio-idUSKBN2AO2OH (accessed 30 Mar 2021).

44 Gholizadeh P, Sanogo M, Oumarou A, *et al.* Fighting COVID-19 in the West Africa after experiencing the Ebola epidemic. *Health Promot Perspect* 2021;**11**:5–11. doi:10.34172/hpp.2021.02

45 Dia N, Lakh NA, Diagne MM, *et al.* COVID-19 Outbreak, Senegal, 2020. *Emerg Infect Dis* 2020;**26**:2771–3. doi:10.3201/eid2611.202615

46 Dong E, Du H, Gardner L. An interactive web-based dashboard to track COVID-19 in real time. *Lancet Infect Dis* 2020;**20**:533–4. doi:10.1016/S1473-3099(20)30120-1

47 Crisis24. Senegal: Authorities reintroduce COVID-19 measures following spike in cases. 2020.https://crisis24.garda.com/alerts/2020/08/senegal-authorities-reintroduce-covid-19-measures-following-spike-in-cases-august-8-update-12 (accessed 28 Jan 2022).

48 Our World in Data. COVID-19 Data Explorer. Our World Data. 2022.https://ourworldindata.org/coronavirus-data-explorer (accessed 28 Jan 2022).

49 Rosenthal PJ, Breman JG, Djimde AA, *et al.* COVID-19: Shining the Light on Africa. *Am J Trop Med Hyg* Published Online First: 5 May 2020. doi:10.4269/ajtmh.20-0380

50 Sougou NM, Diouf J, Leye MMM, *et al.* What community-based public health approaches in West Africa for COVID-19 epidemic? A reflection based on the African socio-cultural context. *Pan Afr Med J* 2020;**35**. doi:10.11604/pamj.supp.2020.35.2.24415

For peer review only

51 Gesesew HA, Koye DN, Fetene DM, *et al.* Risk factors for COVID-19 infection, disease severity and related deaths in Africa: a systematic review. *BMJ Open* 2021;**11**:e044618. doi:10.1136/bmjopen-2020-044618

52 Kana MA, LaPorte R, Jaye A. Africa's contribution to the science of the COVID-19/SARS-CoV-2 pandemic. *BMJ Glob Health* 2021;**6**:e004059. doi:10.1136/bmjgh-2020-004059

For peer review only

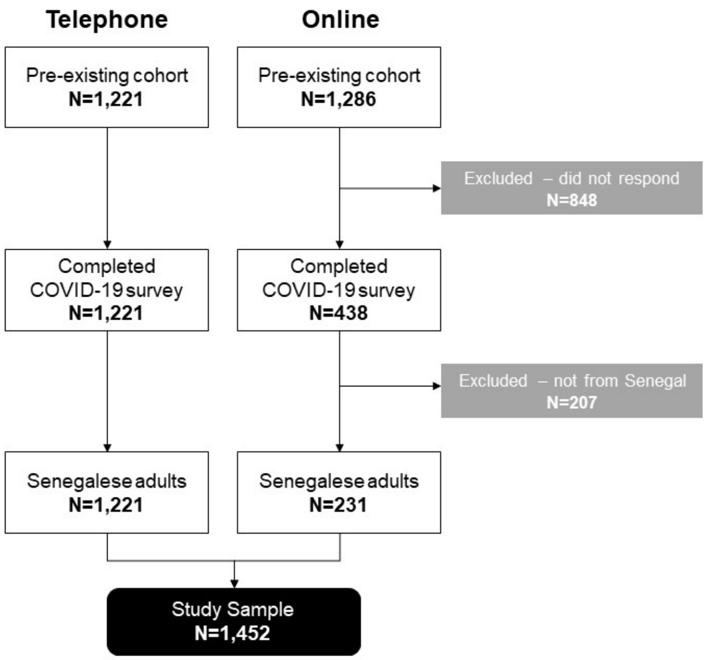

Figure 1. Study recruitment and sampling diagram.

254x190mm (96 x 96 DPI)

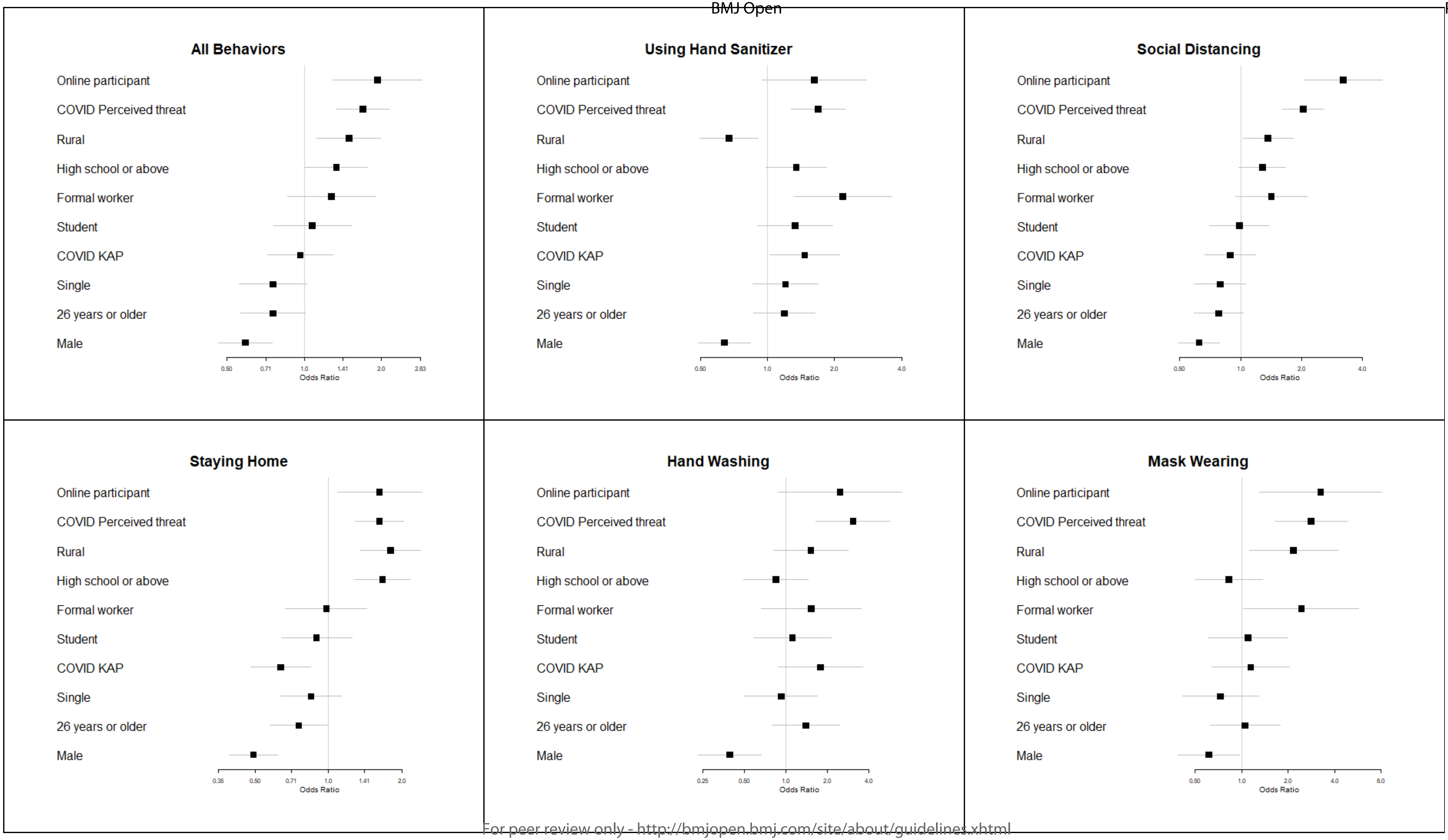
All Behaviors
Online participant
COVID Perceived threat
Rural
High school or above
Formal worker
Student
COVID KAP
Single
26 years or older
Male
0.50
0.71
1.0
1.41
2.0
2.83
Odds Ratio
Using Hand Sanitizer
Online participant
COVID Perceived threat
Rural
High school or above
Formal worker
Student
COVID KAP
Single
26 years or older
Male
0.50
1.0
2.0
4.0
Odds Ratio
Social Distancing
Online participant
COVID Perceived threat
Rural
High school or above
Formal worker
Student
COVID KAP
Single
26 years or older
Male
0.50
1.0
2.0
4.0
Odds Ratio
Staying Home
Online participant
COVID Perceived threat
Rural
High school or above
Formal worker
Student
COVID KAP
Single
26 years or older
Male
0.35
0.50
0.71
1.0
1.41
2.0
Odds Ratio
Hand Washing
Online participant
COVID Perceived threat
Rural
High school or above
Formal worker
Student
COVID KAP
Single
26 years or older
Male
0.25
0.50
1.0
2.0
4.0
Odds Ratio
Mask Wearing
Online participant
COVID Perceived threat
Rural
High school or above
Formal worker
Student
COVID KAP
Single
26 years or older
Male
0.50
1.0
2.0
4.0
8.0
Odds Ratio

Supplemental Table 1. Sample characteristics stratified by recruitment method. N=1,452. Chi-squared and paired t-tests assessed differences between methods. Alpha=0.05. Column percentages presented. Data collected via phone and online surveys from Senegalese adults between June-August 2020.

**Characteristic**	**Category**	**Phone**	**Online**	**Total**	***p***
Age (Mean/Median)		28.5/27.0	25.0/23.0	28.0/26.0	<.001
Age Quartile	<21	23.1	30.7	24.3	<.001
	22-25	22.3	34.6	24.2	
	26-34	23.9	26.8	24.4	
	35+	30.7	7.8	27.1	
Sex	Female	50.1	60.6	51.8	0.003
	Male	49.9	39.4	48.2	
Location	Urban	74.7	89.6	77.1	<.001
	Rural	23.0	4.4	20.1	
	Urban + Rural	2.3	6.1	2.9	
Education	Superior	16.1	79.7	26.2	<.001
	Secondary	25.1	16.0	23.7	
	Primary	23.3	0.0	19.6	
	Middle	18.6	1.7	15.9	
	No	11.8	0.4	10.0	
	Other	5.1	2.2	4.6	
Marital status	Single	50.9	78.8	55.4	<.001
	Married - monogamous	34.9	14.7	31.7	
	Married - polygamist	12.5	3.9	11.1	
	Divorced	1.2	2.2	1.4	
	Widower	0.5	0.4	0.5	
Occupation	Informal sector	45.6	5.2	39.2	<.001
	Student	20.0	67.5	27.6	
	Formal sector	14.6	20.4	15.5	
	Other	8.4	3.9	7.7	
	Housewife	7.5	2.2	6.6	
	Retired, out of work	3.9	0.9	3.4	
	Religious leader	0.1	0.0	0.1	
Religion	Muslim	95.9	87.0	94.5	<.001
	Catholic	3.9	7.8	4.5	
	Protestant / Evangelical	0.1	3.0	0.6	
	No	0.0	1.3	0.2	
	Other	0.1	0.9	0.2	
	Traditionalist	0.1	0.0	0.1	

For peer review only

Supplemental Table 2. Descriptive statistics for novel COVID-19 subscales on knowledge & awareness (n=1,447), perceived threat (n=1,445), prevention behaviors (n=1,447), and all subscales combined (n=1,441). Subscales ranged from 0 [min] to 15 [max]; total scale ranged from 0 to 45. Data collected via phone and online surveys from Senegalese adults between June-August 2020.

	Mean	SD	Median
Knowledge & awareness	11.8	2.6	12.5
Perceived threat	10.9	3.5	11.7
Prevention behaviors	10.5	2.3	11.0
Total	33.1	6.0	34.0

For peer review only

Reporting checklist for cross sectional study.

Based on the STROBE cross sectional guidelines.

Instructions to authors

Complete this checklist by entering the page numbers from your manuscript where readers will find each of the items listed below.

Your article may not currently address all the items on the checklist. Please modify your text to include the missing information. If you are certain that an item does not apply, please write "n/a" and provide a short explanation.

Upload your completed checklist as an extra file when you submit to a journal.

In your methods section, say that you used the STROBE cross sectionalreporting guidelines, and cite them as:

von Elm E, Altman DG, Egger M, Pocock SJ, Gotzsche PC, Vandenbroucke JP. The Strengthening the Reporting of Observational Studies in Epidemiology (STROBE) Statement: guidelines for reporting observational studies.

		Reporting Item	Page Number
**Title and abstract**			
Title	#1a	Indicate the study's design with a commonly used term in the title or the abstract	1

For peer review only

Abstract	#1b	Provide in the abstract an informative and balanced summary of what was done and what was found	2
**Introduction**			
Background / rationale	#2	Explain the scientific background and rationale for the investigation being reported	5-7
Objectives	#3	State specific objectives, including any prespecified hypotheses	7
**Methods**			
Study design	#4	Present key elements of study design early in the paper	8
Setting	#5	Describe the setting, locations, and relevant dates, including periods of recruitment, exposure, follow-up, and data collection	8
Eligibility criteria	#6a	Give the eligibility criteria, and the sources and methods of selection of participants.	8-9
	#7	Clearly define all outcomes, exposures, predictors, potential confounders, and effect modifiers. Give diagnostic criteria, if applicable	10-11
Data sources / measurement	#8	For each variable of interest give sources of data and details of methods of assessment (measurement). Describe comparability of	9-10

For peer review only

		assessment methods if there is more than one group. Give information separately for for exposed and unexposed groups if applicable.	
Bias	#9	Describe any efforts to address potential sources of bias	10-11
Study size	#10	Explain how the study size was arrived at	8-9
Quantitative variables	#11	Explain how quantitative variables were handled in the analyses. If applicable, describe which groupings were chosen, and why	10
Statistical methods	#12a	Describe all statistical methods, including those used to control for confounding	10-11
Statistical methods	#12b	Describe any methods used to examine subgroups and interactions	10-11
Statistical methods	#12c	Explain how missing data were addressed	9
Statistical methods	#12d	If applicable, describe analytical methods taking account of sampling strategy	11
Statistical methods	#12e	Describe any sensitivity analyses	N/A - Compared by recruitment method but didn't look at one specific group.

Results

For peer review only

Participants	#13a	Report numbers of individuals at each stage of study—eg numbers potentially eligible, examined for eligibility, confirmed eligible, included in the study, completing follow-up, and analysed. Give information separately for for exposed and unexposed groups if applicable.	12
Participants	#13b	Give reasons for non-participation at each stage	N/A - Only completed responses were included.
Participants	#13c	Consider use of a flow diagram	9
Descriptive data	#14a	Give characteristics of study participants (eg demographic, clinical, social) and information on exposures and potential confounders. Give information separately for exposed and unexposed groups if applicable.	12-13
Descriptive data	#14b	Indicate number of participants with missing data for each variable of interest	N/A - Only complete responses were included in the study sample.
Outcome data	#15	Report numbers of outcome events or summary measures. Give information separately for exposed and unexposed groups if applicable.	12-14

For peer review only

Main results	#16a	Give unadjusted estimates and, if applicable, confounder-adjusted estimates and their precision (eg, 95% confidence interval). Make clear which confounders were adjusted for and why they were included	14
Main results	#16b	Report category boundaries when continuous variables were categorized	11
Main results	#16c	If relevant, consider translating estimates of relative risk into absolute risk for a meaningful time period	N/A - Results estimates were reported as unadjusted and adjusted odds ratios.
Other analyses	#17	Report other analyses done—e.g., analyses of subgroups and interactions, and sensitivity analyses	12
**Discussion**			
Key results	#18	Summarise key results with reference to study objectives	21
Limitations	#19	Discuss limitations of the study, taking into account sources of potential bias or imprecision. Discuss both direction and magnitude of any potential bias.	27

For peer review only

Interpretation	#20	Give a cautious overall interpretation considering objectives, limitations, multiplicity of analyses, results from similar studies, and other relevant evidence.	27-28
Generalisability	#21	Discuss the generalisability (external validity) of the study results	27
**Other Information**			
Funding	#22	Give the source of funding and the role of the funders for the present study and, if applicable, for the original study on which the present article is based	29

Notes:

- 12e: N/A - Compared by recruitment method but didn't look at one specific group.
- 13b: N/A - Only completed responses were included.
- 14b: N/A - Only complete responses were included in the study sample.
- 16c: N/A - Results estimates were reported as unadjusted and adjusted odds ratios. The STROBE checklist is distributed under the terms of the Creative Commons Attribution License CC-BY. This checklist was completed on January 31, 2022.

For peer review only
